# Urinary Incontinence in Women Athletes: Umbrella Review and Meta-analysis of Sport-Related Factors

**DOI:** 10.1007/s40279-026-02438-z

**Published:** 2026-04-16

**Authors:** Nuria Domínguez-Pérez, Irene Sevilla-Arrabal, Beatriz Navarro-Brazález, María Torres-Lacomba, Javier Courel-Ibáñez

**Affiliations:** 1 Physiotherapy in Women’s Health Research Group, Nursing and Physiotherapy Department, Universidad de Alcalá, Alcalá de Henares, Madrid, Spain; 2 Department of Physical Education and Sport, Faculty of Sport Sciences, University of Granada, Carretera de Alfacar, s/n, 18071 Granada, Spain; 3 Ramón y Cajal Institute of Health Research-IRYCIS, University Hospital of Ramón y Cajal, Madrid, Spain

## Abstract

**Background:**

Urinary incontinence is frequently reported in women athletes, yet prevalence estimates vary widely and are commonly interpreted using broad sport classifications that may not capture pelvic floor loading.

**Objective:**

We aimed to synthesise evidence on urinary incontinence prevalence in adult women athletes and examine whether commonly used sport classifications or reported training exposure explain between-cohort variability.

**Methods:**

Five databases were searched (January 2010–September 2025) for systematic reviews reporting urinary incontinence prevalence in women athletes to conduct an umbrella review. Eligible primary observational studies were identified from included reviews and forward citation tracking to conduct a de novo random-effects meta-analysis and meta-regression restricted to adult women athletes (aged 18–45 years).

**Results:**

Fourteen systematic reviews were included, most rated as low or critically low confidence. Thirty-two primary studies (120 cohorts; *n* = 4649) were meta-analysed, all rated as high or moderate quality. Urinary incontinence prevalence showed substantial between-cohort heterogeneity. In meta-regression analyses, prevalence did not differ significantly across sport discipline, impact category, or sport modality. Weekly training volume was the most consistently reported exposure metric and showed a positive association with urinary incontinence odds (6% per additional hour/week; *p* = 0.011), within the constraints of limited intensity and pelvic floor-specific exposure data, with no evidence of effect modification by impact category or competitive level. Competitive level showed a non-robust directional trend toward higher prevalence among professionals (*p* = 0.08).

**Conclusions:**

Commonly used sport-label classifications showed limited explanatory value for urinary incontinence prevalence in women athletes when applied as stand-alone proxies for exposure, and may misdirect screening and prevention strategies. Within the current heterogeneous and cross-sectional evidence base, cumulative training exposure emerged as the most consistently reported correlate of urinary incontinence prevalence. These findings highlight the need for prospective studies using standardised training metrics with pelvic floor-specific measures to better characterise a multi-dimensional pelvic floor exposure.

**Supplementary Information:**

The online version contains supplementary material available at 10.1007/s40279-026-02438-z.

## Key Points

**Table:** 

Urinary incontinence affects approximately four in ten adult women athletes, but prevalence estimates vary widely between cohorts.
Common sport labels showed limited ability to explain between-cohort variability in urinary incontinence prevalence in meta-regression, likely reflecting exposure misclassification and unmeasured pelvic floor-specific loading.
Sport type-based risk stratification may fail to capture clinically relevant exposure patterns and could delay identification of athletes at risk of urinary incontinence.
Weekly training volume showed the most consistent association with urinary incontinence odds, but should not be interpreted as a mechanistic proxy for pelvic floor loading and instead represents an exposure-based hypothesis-generating indicator.
Clinically, these findings support exposure-aware screening and early care pathways across sports and future research integrating training exposure, pelvic floor-specific measures and symptom response to characterise a multi-dimensional pelvic floor “dose”.

## Introduction

Pelvic floor disorders (PFD), particularly urinary incontinence (UI), affect a substantial proportion of women and can influence daily functioning, participation and sport performance [1, 2]. Although age and parity are established risk factors [3, 4], up to one in five nulliparous women of reproductive age report UI [5], indicating contributors beyond obstetric history. In sport, UI is frequently described among women athletes and may be normalised or under-reported despite potential impacts on women’s health and sports careers [6, 7]. Over time, unaddressed pelvic floor symptoms may impair quality of life, hinder athletic performance [8] and contribute to premature dropout from sport [9]. Ultimately, the culture of high-performance sport, characterised by physical intensity, performance pressure and limited pelvic health education, may perpetuate the normalisation and under-reporting of UI among women athletes, delaying timely support and intervention [10, 11]. Against this backdrop, interest has grown in sport-related determinants of pelvic floor health, including mechanical loading, neuromuscular fatigue and training demands [12, 13].

Previous systematic reviews and meta-analyses consistently report that UI is common among physically active women and athletes; however, translating these findings into athlete care remains limited by substantial methodological heterogeneity. Reviews frequently combine populations with differing competitive contexts, training exposures, parity status and sport-specific demands, complicating the interpretation of prevalence estimates. For instance, some syntheses restrict inclusion to nulliparous women [14, 15], whereas others pool nulliparous and parous athletes across wide age ranges, despite parity and age being strong confounders of UI risk [16, 17]. Similarly, elite competitors are frequently combined with recreational exercisers without explicit thresholds for training exposure, competitive level, or performance context [18–20]. Even within sport-specific analyses, samples often encompass wide variation in parity, training dose and experience, constraining inference regarding sport-level risk patterns [21–24]. As a result, reported prevalence estimates vary widely across syntheses, even within ostensibly similar sport categories, limiting comparability and leaving clinicians and performance staff without athlete-specific prevalence estimates to guide screening and prevention strategies.

Importantly, many syntheses have treated sport type or impact classification as key determinants of UI prevalence across athletic populations [6, 21], yet the extent to which these commonly used classifications actually explain between-cohort variability in prevalence has rarely been examined quantitatively. This reliance on nominal sport classifications assumes that sport type meaningfully reflects pelvic floor loading. Although specific high-impact tasks (e.g. jumping and landing activities) are recognised to provoke leakage in certain athletic contexts [6, 12], it remains uncertain whether broad sport labels independently explain between-cohort variability in UI prevalence when examined quantitatively. Such classifications may therefore incompletely capture how pelvic floor stress accumulates across training volume, intensity and recovery patterns in real-world sport participation [25, 26]. When sport impact is defined strictly according to primary studies’ classifications, comparisons between high-impact and low-impact activities are often based on a small number of cohorts [27]. Conversely, broader post hoc sport discipline classifications may attenuate apparent differences between impact categories because of substantial heterogeneity and potential exposure misclassification [20]. Consequently, the field lacks a robust quantitative approach to characterising pelvic floor loading during sport participation, and current interpretations may overemphasise nominal sport labels while overlooking cumulative training exposure and contextual sport factors.

Collectively, these limitations constrain the interpretability and generalisability of existing syntheses and highlight the difficulty of identifying modifiable sport-related factors relevant to pelvic floor health. Contemporary consensus statements and female athlete health frameworks emphasise the need for higher-quality context-specific evidence to inform screening, education and care pathways in high-performance environments [25]. In this context, an umbrella review alone would summarise inconsistent findings without resolving athlete-specific questions or empirically testing whether commonly used sport classifications meaningfully explain variability in UI prevalence across cohorts. Accordingly, this study was designed as an overview of reviews incorporating a de novo systematic review and meta-analysis of eligible primary cohorts, consistent with Cochrane guidance on overviews and outcome-centric synthesis [28–30]. This combined approach allows critical appraisal of the existing review literature while providing a more coherent synthesis of sport-related factors relevant to women athletes.

The aims of this umbrella review and meta-analysis were therefore to (1) synthesise and update evidence on the prevalence of UI among adult women athletes, and (2) examine how sport context (discipline, modality and competitive level), commonly used mechanical load classifications (sport impact) and cumulative exposure (training volume) contribute to between-cohort variability in pelvic floor symptom prevalence.

## Methods

For the umbrella review, we identified and screened systematic reviews and meta-analyses according to predefined eligibility criteria focused on UI prevalence in physically active or athletic women populations. From eligible reviews, we extracted the cited primary observational studies reporting prevalence data. To ensure a comprehensive and updated synthesis of the available evidence, we also applied a snowball search strategy (forward citation tracking) to locate additional updated primary studies not captured in the original systematic reviews. For the meta-analysis, all primary studies meeting the inclusion criteria were subjected to independent quality appraisal and then included in a new meta-analysis to generate updated prevalence estimates. This dual-layered approach allowed us to summarise the synthesised evidence while expanding and refining it using original primary data. The study forms part of a larger overview project exploring the prevalence of PFD in nulliparous women athletes. The protocol was prospectively registered in PROSPERO (#CRD420251055357) and follows the Preferred Reporting Items for Systematic Reviews and Meta-Analyses (PRISMA) reporting guidelines [31, 32].

### Literature Search

Records were retrieved from five electronic databases: MEDLINE (via PubMed), Scopus, Web of Science, CINAHL (via EBSCOhost) and the Cochrane Library. The search was limited to reviews published from January 2010 to 29 September, 2025. Search strategies for each database combined Medical Subject Headings (or equivalent) and relevant free-text terms related to UI, athletic populations and prevalence. Forward citation tracking (via Google Scholar) was manually screened on all included systematic reviews to identify additional primary studies. The full search strategy was developed as part of a broader overview-of-reviews project on the prevalence of PFD in women athletes and is available in Appendix 1 of the Electronic Supplementary Material (ESM). Analyses were restricted to adult women athletes (aged 18–45 years) to reduce developmental and hormonal heterogeneity, as adolescence and perimenopause represent distinct life stages with respect to pelvic floor health and training exposure [25].

### Eligibility Criteria for Systematic Reviews and Meta-analyses

#### Study Population

Adult women athletes aged 18–45 years. Para athletes were excluded.

#### Exposure

Engagement in regular sport participation (training and/or competition), irrespective of sport modality or performance level.

#### Outcomes and Instruments

Prevalence of any type of UI, (overall UI, stress UI [SUI], urgency UI [UUI], mixed UI [MUI] and exercise-related/exertional UI [EXUI]), assessed using any measurement approach (e.g. validated questionnaires, clinical assessment, self-report).

#### Study Design

Systematic reviews or meta-analyses.

#### Publication Status, Language Restrictions and Timeframe

Studies published in peer-reviewed academic journals, in English, French, Portuguese or Spanish (reflecting the language proficiency of the review team), published after 1 January, 2010 to reduce redundancy and excessive overlap. Conference abstracts, theses and non-peer-reviewed reports were excluded.

### Eligibility Criteria for Primary Studies

#### Study Population

Adult women athletes aged 18–45 years. Para athletes were excluded.

#### Exposure

Participation in a specific sport discipline (e.g. soccer, CrossFit, rugby, cycling). Studies were included when the sport discipline was clearly reported, even if the specific specialty was not specified (e.g. athletics or track and field, gymnastics, dance). Studies were excluded when sport participation was reported only at a broad or non-specific level (e.g. collective sports, endurance sports, or physically active) without discipline-level classification, as such classifications were not sufficiently granular for quantitative synthesis.

#### Outcomes and Instruments

Prevalence of any type of UI, eligible for quantitative synthesis if measured with (a) validated questionnaires, (b) clinician-administered structured assessments (e.g. standardised clinical screening, pad tests) or (c) clearly defined self-report items aligned with the International Continence Society definitions (with an explicit recall period). Studies using lifetime/unspecified recall or not validated questionnaires were included but flagged and examined in sensitivity analyses. Where available, we extracted subtype-specific prevalence (SUI, UUI, MUI and EXUI).

#### Study Design

Prospective or retrospective cohorts, case–control, cross-sectional or intervention studies.

#### Publication Status, Language Restrictions and Timeframe

Studies published in peer-reviewed academic journals, in English, French, Portuguese or Spanish (reflecting the language proficiency of the review team), with no restriction on year of publication. Conference abstracts, editorials, commentaries, dissertations and other forms of grey literature were excluded.

### Study Selection

Data management was conducted using the systematic review management software Rayyan. Duplicate entries were identified and removed using Rayyan’s semi-automated duplicate detection tool, followed by manual verification to ensure accuracy. Two reviewers (ISA and NDP) independently screened titles and abstracts against predefined inclusion and exclusion criteria. Full texts were then retrieved and assessed for eligibility. Disagreements at any stage were resolved through discussion or, if needed, by a third reviewer (JCI). Additional studies identified through forward citation tracking were screened using the same process.

### Data Extraction

A standardised data extraction form was developed a priori and piloted on a subset of studies to ensure clarity and consistency. Data extraction was independently performed by two reviewers (ISA and NDP), with disagreements resolved through discussion or, if needed, adjudicated by a third reviewer (JCI). Authors were contacted to clarify missing or unclear data when necessary.

For systematic reviews and meta-analyses included in the umbrella review, extracted data comprised study characteristics (authors, year and design), population characteristics (number of included studies, sample size, age range, parity status, level of competition and sport discipline/exposure, when reported), and UI-related outcomes. Reviews that did not restrict inclusion criteria by parity status, competitive level or sport discipline/exposure were classified as mixed.

For primary studies included in the meta-analysis, extracted data included study characteristics (authors, year, country, design), participant characteristics (sample size, age, parity status, race/ethnicity and socioeconomic status), exposure details (sport discipline, sport modality, competitive level), weekly training volume, UI instruments and quantitative prevalence (events and total sample). The primary outcome variable was UI and UI subtype prevalence, extracted as the number of UI events and total sample size. Instruments were coded as Validated (named, referenced tool) versus International Continence Society Criteria aligned item (single/multi-item, not validated, maps to International Continence Society Criteria ICS definition) for sensitivity analyses. Exposure and classification variables extracted or derived for analysis included were classified as follows: (1) Sport discipline, as reported in the original study; (2) Sport modalities, defined as groups of sport disciplines sharing common biomechanical and physiological characteristics and classified into Technical, Endurance, Aesthetic, Weight-dependent, Ball Games, Power and Gravity sports [33]; (3) Sport impact, based on the mechanical load transferred to the pelvic floor through increased intra-abdominal pressure and ground reaction forces, according to pelvic floor-specific frameworks and consensus [34, 35]: High-impact sports (e.g. gymnastics, basketball, volleyball, high jump, trampoline, powerlifting), Medium-impact sports (e.g. tennis, running, karate, soccer), Low-impact sports (e.g. swimming, cycling and walking, where pelvic floor strain is minimised because of limited ground impact or absence of explosive movements); (4) Competitive level, conceptualised as a proxy for long-term cumulative exposure and training context rather than performance status alone, based on training volume, performance level and competitive context [36]: Amateurs (individuals engaged in structured training without elite or national-level competition), High-level group (athletes competing at the national level with structured training and competition schedules; this category also included athletes with at least 3 years of sub-elite competitive involvement), and Professionals (athletes competing at the highest national or international levels, training in elite or Olympic centres or explicitly identified as elite by the original study authors); and (5) Training volume, extracted as mean or median hours per week as the central tendency [37].

### Quality Assessment

The methodological quality of included systematic reviews and meta-analyses was evaluated using the AMSTAR 2 (A Measurement Tool to Assess Systematic Reviews) instrument [38]. AMSTAR 2 evaluates 16 methodological domains, including protocol registration, comprehensiveness of the literature search, duplicate study selection and data extraction, risk-of-bias assessment of primary studies, appropriateness of meta-analytic methods and consideration of risk of bias when interpreting results. Overall confidence in review findings was determined based on the presence and severity of methodological limitations across domains, classified into three categories: Non-critical weaknesses: Items 1, 3, 5, 6, 8, 10, 14 and 16, Critical weaknesses: Items 2, 4, 7, 11 and 15, and Critical flaws: Items 9, 12 and 13. Reviews presenting one or more critical flaws were rated as having critically low confidence. Reviews without critical flaws but presenting at least one critical weakness were rated as having low confidence. Reviews with no critical weaknesses or flaws were initially rated as having moderate confidence; however, the presence of multiple non-critical weaknesses was considered sufficient to further downgrade confidence from moderate to low when appropriate. Reviews with no or only one non-critical weakness were rated as having high confidence.

For primary studies included in the meta-analysis, methodological quality was evaluated using the Appraisal Tool for Cross-sectional Studies (AXIS) [39], a 20-item checklist developed for observational research. AXIS assesses three broad domains: reporting quality (clarity of objectives, methods and results), study design quality (sampling procedures, sample size justification and handling of non-responders) and risk of bias (validity of outcome measurement, consideration of confounding and appropriateness of statistical analyses). Item fulfilment was coded as Yes (1 point), Partial (0.5 points) or No (0 points), and total AXIS scores were used to classify studies as high quality (≥ 16.5 points), moderate quality (13.0–16.0 points) or low quality (< 13.0 points).

Small-study effects were assessed when *k* ≥ 10 using funnel plots and Egger’s test. To avoid double-counting of primary studies, we identified systematic reviews with non-overlapping evidence for each outcome. When multiple reviews were available, we calculated the corrected covered area to quantify overlap [40]. If overlap was high (corrected covered area > 10%), older or lower-quality reviews were excluded to ensure that each primary study contributed only once to the synthesis.

### Statistical Analysis

Each study was rated independently by two reviewers (ISA, NDP), with disagreements resolved through discussion (JCI). Data from primary studies were aggregated to one cohort per study before pooling (*k* denotes cohorts). Random-effects models pooled logit-transformed prevalence (PLOGIT) with 95% confidence intervals (CIs) and 95% prediction intervals; heterogeneity was quantified by *τ*^2^, *I*^2^ and Cochran’s *Q*. Pre-specified subgroup analyses compared sport discipline, modality, impact and competitive level; only subgroups with three or more cohorts were meta-analysed. Subgroup differences were tested with the *χ*^2^ test for subgroup differences (unadjusted comparisons of pooled subgroup means). We also fitted random-effects meta-regressions with moderators entered categorically (QM). Because these approaches use different models and weighting, they may yield different inferences; meta-regression was considered primary. Pairwise comparisons used re-levelling, reported as log-odds with 95% CIs and odds ratios (ORs). Moderator pseudo-*R*^2^ was the proportional reduction in *τ*^2^ versus the null model. Training volume was entered as continuous predictors, reported as OR per 1 standard deviation (SD) and per unit (h·week⁻^1^). Egger’s test was applied only where *k* ≥ 10. Analyses were run in R (Version 4.1.2).

### Sensitivity Analysis

To verify the robustness of pooled and subgroup estimates, we re-ran the entire analysis pipeline under two prespecified restrictions: (1) validated instruments only, retaining studies that explicitly used standardised UI tools (e.g. ICIQ-UI SF, UDI-6), and (2) nulliparous samples only, retaining studies that reported data exclusively for nulliparous women. Across sensitivity runs, we report whether the direction and magnitude of the global prevalence, subgroup contrasts and continuous associations remained consistent with the primary analyses.

## Results

### Umbrella Review

#### Study Selection

**Fig. 1:**
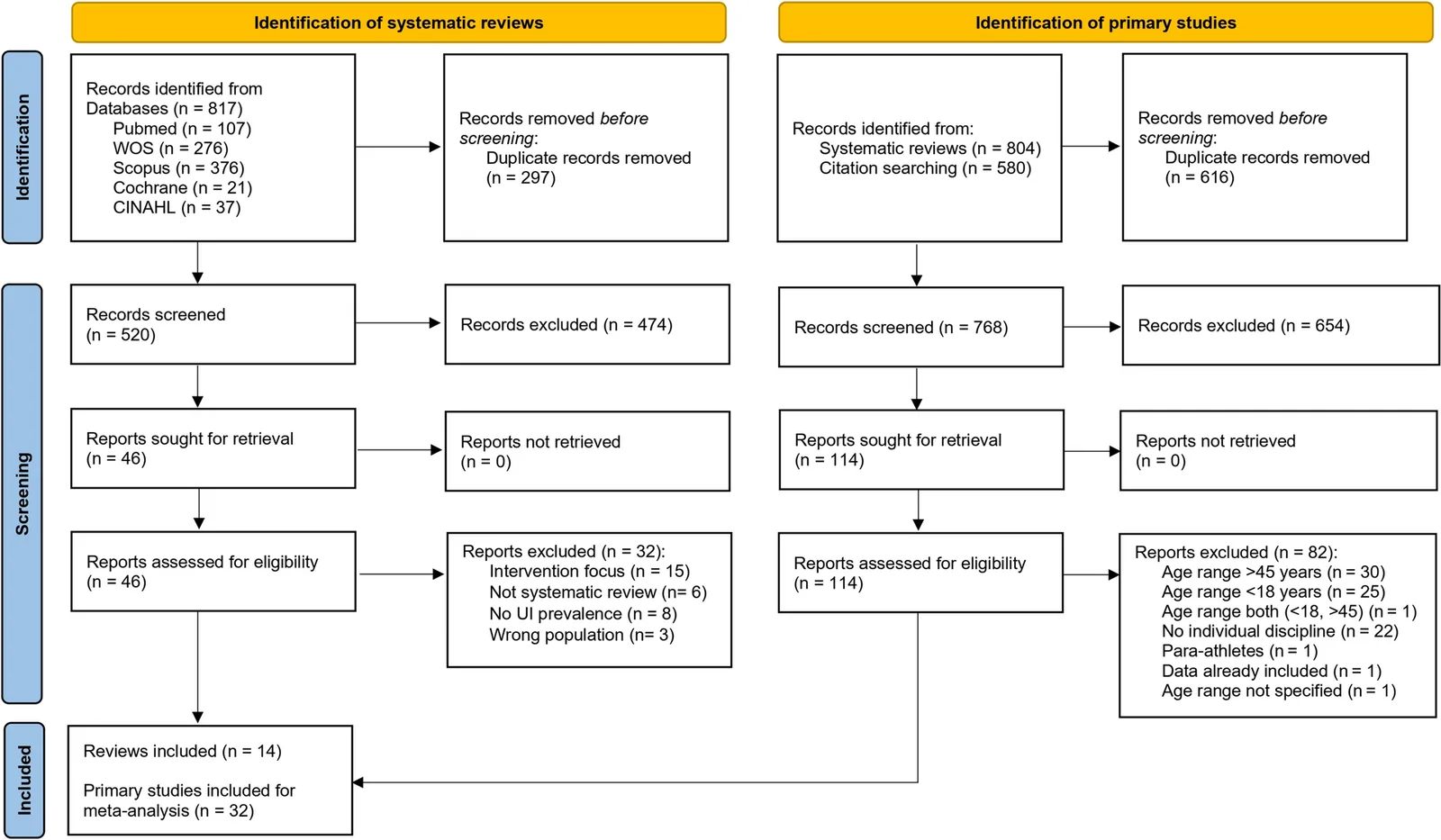
Preferred Reporting Items for Systematic Reviews and Meta-Analyses (PRISMA) flow diagram illustrating the number of records identified, screened, assessed for eligibility and included in the review. A full list of excluded studies with reasons is available in Appendices 2 and 5 of the ESM. *UI* urinary incontinence, *WOS* Web of Science

A total of 817 systematic reviews were identified from the database searches. After removing duplicates and screening titles and abstracts, 46 systematic reviews remained for further full-text screening. Following a careful full-text screening, 14 systematic reviews were included in the umbrella review [14–19, 21–24, 27, 41, 42] (Fig. 1). A full list of excluded studies with reasons is available in Appendix 2 of the ESM.

#### Quality Assessment

AMSTAR 2 classified 12 of 14 included reviews as critically low confidence, and two were rated low confidence (Appendix 3 of the ESM). The most frequent critical issues were the absence of a pre-registered protocol or a priori design (Item 2), and the failure to provide a list of excluded studies with justification (Item 7), which was absent in all reviews. Additional recurring limitations included inadequate or non-validated risk-of-bias assessment of primary studies (Item 9) [14–18, 41, 42], failure to consider risk of bias when interpreting pooled results (Item 13) [14, 17, 18, 41], and lack of publication bias assessment when a meta-analysis was conducted and appropriate (Item 15) [14, 20, 24, 27]. Across 14 systematic reviews comprising 99 unique primary studies, the corrected covered area was 6.5%, indicating a moderate overlap and supporting the retention of all eligible systematic reviews. The citation matrix used to assess overlap is provided in Appendix 4 of the ESM.

#### Study Characteristics

The umbrella review included eight systematic reviews and six meta-analyses published between 2017 and 2025, encompassing populations across a wide age spectrum and frequently combining adolescent and adult samples, with heterogeneous parity status reported (Table 1). Only three reviews restricted inclusion to nulliparous women [14, 15, 19]. Competitive level was inconsistently reported and commonly aggregated across amateur, high-level and professional athletes. Only two reviews specifically addressed professional athletes [41, 42]. The scope of the included reviews varied considerably, with qualitative syntheses that ranged from < 5 to > 30 primary studies. Sport classification across reviews was heterogeneous, with most reviews examining mixed sports. Some reviews focused on specific disciplines, particularly volleyball [42], CrossFit [17, 22, 24] and strength-based sports such as powerlifting and weightlifting [23], whereas others adopted broader classifications based on mechanical impact [18, 20, 21]. Outcomes also varied, with a small number of reviews distinguishing between UI subtypes [16, 20, 22, 41, 42].

**Table 1:** Characteristics of identified systematic reviews and meta-analyses for the umbrella review

First author, year	Design	Age range (years)	Parity status	Level of competition	*n* studies^b^	*n* participants	AMSTAR 2 rating^c^	Sport classification	Outcome	UI prevalence (%)
Almousa, 2019 [14]	SR	12–45	Nulliparous	Mixed	23	2459	Critically low	Mixed sports	UI	5.7–80.0
Álvarez-García, 2022 [22]	SRMA	12–45	Mixed	Mixed	13 (12)	2187	Critically low	CrossFit	UI	32.1 (95% CI 22.2–43.8)
								CrossFit	SUI	35.8 (95% CI 19.4–56.4)
Alves, 2025 [23]	SR	20–79	Mixed	Mixed	5	1809	Low	Powerlifting	UI	41.0–48.8
								Weightlifting	UI	36.6–54.1
Cerruto, 2020 [16]	SRMA^a^	15–> 45	Mixed	Mixed	31 (5)	8335	Critically low	Mixed sports	UI	58.7
								Mixed sports	UI (daily life)	32.8
								Mixed sports	EXUI	36.3
								Mixed sports	SUI	23.0
								Mixed sports	UUI	11.0
								Mixed sports	MUI	11.9
Culleton-Quinn, 2022 [41]	SR	12–89	Mixed	Professional	32	NR	Critically low	Mixed sports	UI, SUI, UUI, MUI	NR
de Mattos Lourenco, 2017 [18]	SR	12–69	Mixed	Mixed	22	7507	Critically low	High-impact	UI	42.2–61.2
								Medium-impact	UI	6.3–44.4
								Low-impact	UI	5.6–15.3
Domínguez-Antuña, 2023 [17]	SRMA^a^	18–71	Mixed	Mixed	13 (13)	4823	Critically low	CrossFit	UI	44.5
								CrossFit	SUI	81.2
García-Perdomo, 2022 [27]	SRMA^a^	17–43	Mixed	Mixed	3 (3)	1018	Critically low	High-impact	UI	12.2–52.9
								Low-impact	UI	3.8–37.5
Martins, 2017 [15]	SR	12–45	Nulliparous	Mixed	10	2272	Critically low	Mixed sports	UI	12.0–80.0
Pires, 2020 [21]	SRMA	18–45	Mixed	Mixed	9	1254	Low	High-impact	UI	25.9 (95% CI 23.5–28.3)
								High-impact	SUI	20.7 (95% CI 18.5–22.9)
Romero Parra, 2024 [24]	SR	18–48	Mixed	Mixed	7	1333	Critically low	CrossFit	UI	32.1–44.5
Silva Pereira, 2017 [42]	SR	NR	Mixed	Professional	5	237	Critically low	Volleyball	EXUI	9.0–30.0
								Volleyball	UI (daily life)	17.0–18.0
Syeda, 2024 [19]	SR	18–45	Nulliparous	Mixed	9	NR	Critically low	Mixed sports	UI	5.7–80.0
Teixeira, 2018 [20]	SRMA	NR	Mixed	Mixed	8 (8)	1714	Critically low	Mixed sports	UI	36.1 (95% CI 26.5–46.8)
								Mixed sports	SUI	43.6 (95% IC 24.4–44.7)
								High-impact	UI	39.9 (95% IC 34.5–45.2)
								Low-/moderate-impact	UI	44.1 (95% IC 36.1–52.3)

*CI* confidence interval, *EXUI* exercise-related/exertional UI, *IC*, *MA* meta-analysis, *MUI* mixed UI, *NR* not reported, *SR* systematic review, *SUI s*tress UI, *UI* urinary incontinence, *UUI* urgency UI

^a^These meta-analyses did not pool UI prevalences

^b^Values are presented as total included studies in the qualitative synthesis (studies contributing to the meta-analysis)

^c^Details on the AMSTAR 2 assessment are available in Appendix 4 of the ESM

#### Urinary Incontinence (UI) Prevalence in Women Athletes Across Systematic Reviews and Meta-analyses

Characteristics of the systematic reviews and meta-analyses are detailed in Table 1. Urinary incontinence was consistently identified as a prevalent condition among sportswomen, although reported prevalence varied widely from 5.7 to 80.0% according to sport modality, mechanical impact profile, population composition and outcome definition. Across UI subtypes, SUI was consistently reported as the predominant, whereas UUI and MUI were less frequently observed.

Stratified findings by sport impact [14, 18, 20, 21, 27] generally reported higher UI prevalence in high-impact sports (12.2–61.2%) compared with low-impact sports (3.8–37.5%). However, meta-analyses pooling single-arm UI prevalence estimates reported overlapping pooled values across impact categories, including 36.1% (95% CI 26.5–46.8) in mixed-sport female athlete populations [20], 44.1% (95% CI 36.1–52.3) in low-impact sports [20], and 25.9% (95% CI 23.5–28.3) and 39.9% (95% CI 34.5–45.2) in high-impact sports [20, 21].

Four reviews were focused on particular sports disciplines. In CrossFit, the overall UI and SUI prevalence of 44.5% and 81.2% [17] and pooled prevalences of UI and SUI of 32.1% (95% CI 22.2–43.8) and 35.8 (95% CI 19.4–56.4) [22], with leakage commonly associated with jump-based exercises (e.g. jump rope single/double unders, box jumps), running and high-load lifts, including deadlifts and squats. In powerlifting and weightlifting, reported UI prevalences were 41.0–48.8% and 36.6–54.1%, with leakage frequently associated with high-load lifts such as squats and deadlifts [23]. In professional volleyball, overall UI prevalence was 9–30% during sports practice and identified urinary leakage as occurring particularly during jumping actions, effort-based and abdominal training, and around competition periods, with higher training and competition exposure associated with more frequent leakage episodes [42].

Conclusions from some reviews were strongly influenced by population composition, particularly age, parity status and competitive exposure. Reviews restricted to nulliparous athletes reported wide prevalence ranges (up to 80%) [14, 15, 19]; however, the highest estimates derived from adolescent and young elite trampolinists in whom involuntary UI was reported during trampoline training [43]. When attention was restricted to adult nulliparous cohorts, UI prevalence estimates were substantially lower and broadly comparable to those reported in parous female athletes, typically with a range from approximately 10–40%. Reviews including mixed parity profiles showed similarly heterogeneous prevalence estimates, reflecting the combined influence of sport-specific mechanical demands and age-related exposure patterns, rather than parity status alone.

Across the included systematic reviews and meta-analyses, several recurrent methodological limitations were consistently highlighted: (1) substantial heterogeneity in sport exposure, outcome definitions and population characteristics, limiting the attribution of UI prevalence to specific sport characteristics; (2) athlete samples frequently pooled multiple disciplines with different impact and load profiles, while sport classification, parity status, age range, competitive level and training volume were inconsistently defined or reported; and (3) the predominance of cross-sectional designs and reliance on self-reported UI further constrained causal inference and increased susceptibility to reporting bias.

### Meta-analysis

#### Study Selection

A total of 1384 primary studies were identified (804 from the reference list and 580 from the forward citation searching). After duplication and title/abstract screening, 114 underwent a full-text review, of which 32 studies [44–76] met the inclusion criteria and were included for quantitative synthesis (Fig. 1). A full list of excluded studies with reasons is available in Appendix 5 of the ESM.

#### Quality Assessment

AXIS classified 14 of 32 original studies as high quality and 18 as moderate quality (Appendix 6 of the ESM). Egger’s regression test did not indicate small-study effects for competitive level (*p* = 0.206), sport impact (*p* = 0.592) or sport modality (*p* = 0.216). However, evidence of funnel plot asymmetry was observed for sport discipline (*p* = 0.043), suggesting that smaller studies within specific sport disciplines tend to report systematically different UI prevalence estimates compared with larger studies. Egger’s regression test and funnel plots for all subgroup meta-analyses are shown in Appendix 7 of the ESM.

#### Study Characteristics

Characteristics of the primary studies are detailed in Table 2. Detailed sport characteristics, UI outcomes and training exposure for each cohort are provided in Appendix 8 of the ESM. Urinary incontinence was primarily assessed using validated self-report questionnaires, most commonly the ICIQ-UI Short Form. Only four studies relied on International Continence Society Criteria-aligned single-item measures and were examined in sensitivity analyses [44, 62, 63, 67]. The included studies comprised a total of 4649 women athletes from 120 cohorts, predominantly nulliparous (93.9% in average). The evidence base was unevenly distributed across sport contexts. Most participants were drawn from ball games and weight-based sports, and the majority competed in high-impact or medium-impact modalities, with low-impact sports under-represented. Competitive level was variably reported, with most cohorts comprising amateur or professional athletes. Overall, the distribution of disciplines and exposure categories highlights substantial clustering of evidence in a limited number of sports, which should be considered when interpreting subgroup analyses.

**Table 2:** Characteristics of included primary studies for the meta-analysis

First author, year	Country	Nulliparous (%)	Age, years, mean^a^	*n* (with UI)	UI assessment (recall window)	AXIS rating^b^
Araujo, 2019 [59]	Brazil	80.2	30.2 (4.6)	127 (45)	ICIQ-UI SF (4 weeks)	Moderate
Cardoso, 2018 [60]	Brazil	100	21.6 (2.7)	118 (82)	ICIQ-UI SF (4 weeks)	High
Caylet, 2006 [44]	France	NR	23.4 (4.5)	126 (32)	ICS-aligned item (days to months)	High
Clapin, 2025 [45]	France	96	24.0 (4.3)	159 (79)	ICIQ-UI SF (4 weeks)	High
Culleton-Quinn, 2024 [46]	Ireland	95	23.4 (2.6)	159 (98)	ICIQ-UI SF (4 weeks)	High
Cygańska, 2025 [47]	Poland	100	24.7 (4.0)	48 (9)	APFQ (unspecified)	High
da Silva Borin, 2013 [48]	Brazil	100	24.0 (8.5)	30 (5)	BFLUTS (unspecified)	Moderate
Da Silva Pereira, 2021 [50]	Brazil	76	25.5	75 (53)	UDI-6SF (unspecified)	Moderate
Dakic, 2025 [61]	Australia	89.3	26.4 (5.0)	28 (13)	ICIQ-UI SF (4 weeks)	High
de Souza Pereira 2022 [49]	Brazil	77.8	30.1	189 (73)	ICIQ-UI SF (4 weeks)	High
Dockter, 2007 [62]	USA	100	19.2 (1.0)	109 (51)	ICS-aligned item (lifetime)	Moderate
dos Santos, 2009 [63]	Brazil	100	21.4 (1.7)	91 (10)	ICS-aligned item (lifetime)	Moderate
Faulks, 2021 [64]	Australia	86.0	26.0 (5.1)	65 (39)	QUID (2 weeks)	Moderate
Fozzatti, 2012 [51]	Brazil	100	25.7 (5.3)	600 (89)	ICIQ-UI SF (4 weeks)	Moderate
Gan, 2025 [65]	USA	100	20.0	29 (18)	LURN SI-29 (week to year)	Moderate
Gill, 2025 [66]	Australia	100	25.0	81 (44)	QUID (lifetime)	High
Hagovska, 2018 [74]	Slovak Republic	100	21.1 (3.9)	278 (33)	ICIQ-UI SF (4 weeks)	High
Jácome, 2011 [67]	Portugal	90.6	23.0 (4.4)	74 (34)	ICS-aligned item (lifetime)	Moderate
Kelečić, 2023 [52]	Croatia	97	22.6 (4.6)	88 (35)	ICIQ-UI SF (4 weeks)	Moderate
Lopes, 2020 [53]	Brazil	94	28.6 (4.5)	50 (10)	ICIQ-UI SF (4 weeks)	High
Machado, 2021 [54]	Brazil	100	27.4 (3.7)	20 (12)	ICIQ-UI SF (4 weeks)	Moderate
McCarthy-Ryan, 2024 [73]	UK	65.7	28.0 (8.0)	396 (250)	ICIQ-UI SF (4 weeks)	Moderate
Middlekauff, 2016 [55]	USA	100	26.8 (3.8)	35 (9)	EPIQ (not reported)	Moderate
Parr, 2023 [56]	USA	100	20.2 (1.5)	202 (68)	ICIQ-UI SF (4 weeks)	High
Patrizzi, 2014 [68]	Brazil	100	23.9 (3.8)	108 (46)	ICS-aligned item (unspecified)	Moderate
Poświata, 2014	Poland	76	28.1 (5.2)	112 (67)	UDI-6 (unspecified)	Moderate
Pisani, 2022 [75]	Brazil	100	28.3 (5.9)	616 (200)	ICIQ-UI SF (4 weeks)	Moderate
Sandwith, 2021 [57]	Canada	100	19.9 (1.8)	95 (51)	UDI-6 (unspecified)	High
Sebastián-Rico, 2024 [69]	Spain	100	25.7 (4.7)	235 (82)	ICIQ-UI SF (4 weeks)	High
Tarczewska, 2024 [70]	Poland	100	22.0	61 (27)	UDI-6SF (unspecified)	Moderate
Valastro, 2025 [71]	Belgium	100	25.0	37 (10)	USP (4 weeks)	High
Winder, 2023 [58]	USA	87.5	25.4 (5.2)	208 (60)	ICIQ-UI SF (4 weeks)	Moderate

*APFQ* Australian Pelvic Floor Questionnaire, *BFLUTS* Bristol Female Lower Urinary Tract, Symptoms Questionnaire, *EPIQ* Epidemiology of Prolapse and Incontinence Questionnaire, *ICIQ* International Consultation on Incontinence Questionnaire, *ICS* International Continence Society Criteria, *LURN SI-29* Symptoms of Lower Urinary Tract Dysfunction Network-Symptom Index 29, *QUID* Questionnaire for Urinary Incontinence Diagnosis, *UI* urinary incontinence, *UDI-6* Urogenital Distress Inventory, *USP* Urinary Symptom Profile

^a^Data are mean (standard deviation) when available

^b^Details on the AXIS assessment are available in Appendix 6 of the ESM

#### Meta-analysis and Meta-regression of UI Prevalence in Women Athletes

**Fig. 2:**
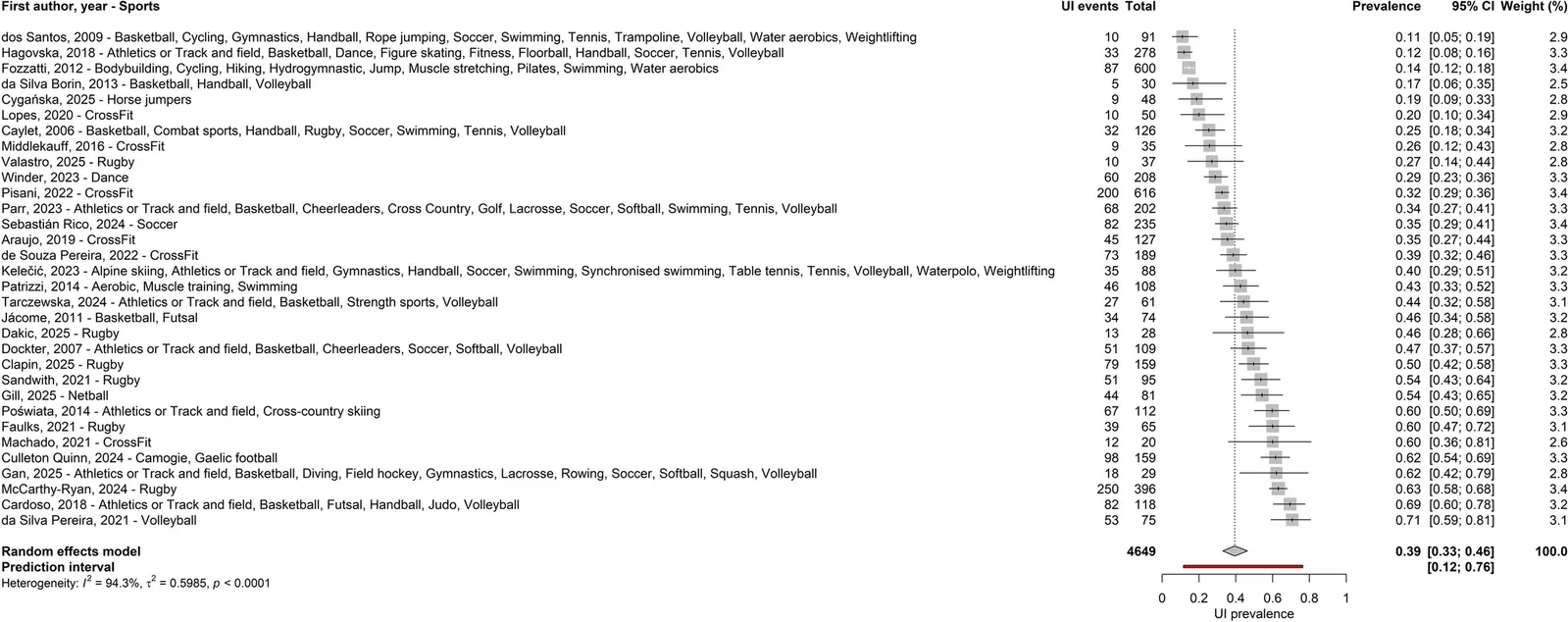
Forest plot of the pooled prevalence of urinary incontinence (UI) in women athletes aged 18–45 years. *CI* confidence interval

**Fig. 3:**
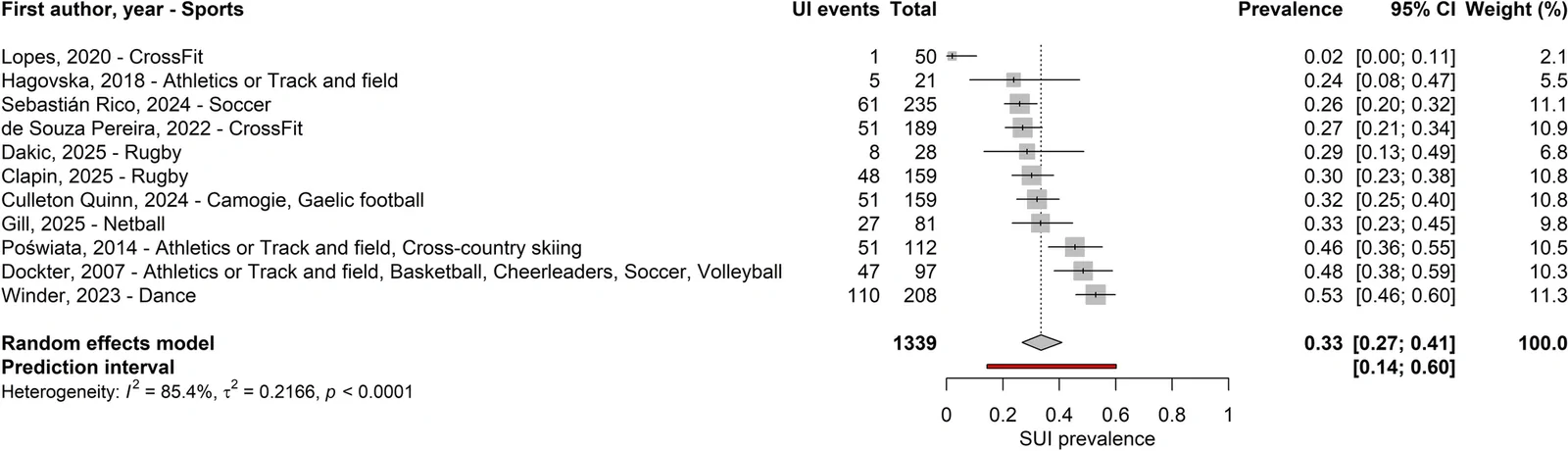
Forest plot of the pooled prevalence of stress urinary incontinence (SUI) in women athletes aged 18–45 years. *CI* confidence interval

**Fig. 4:**
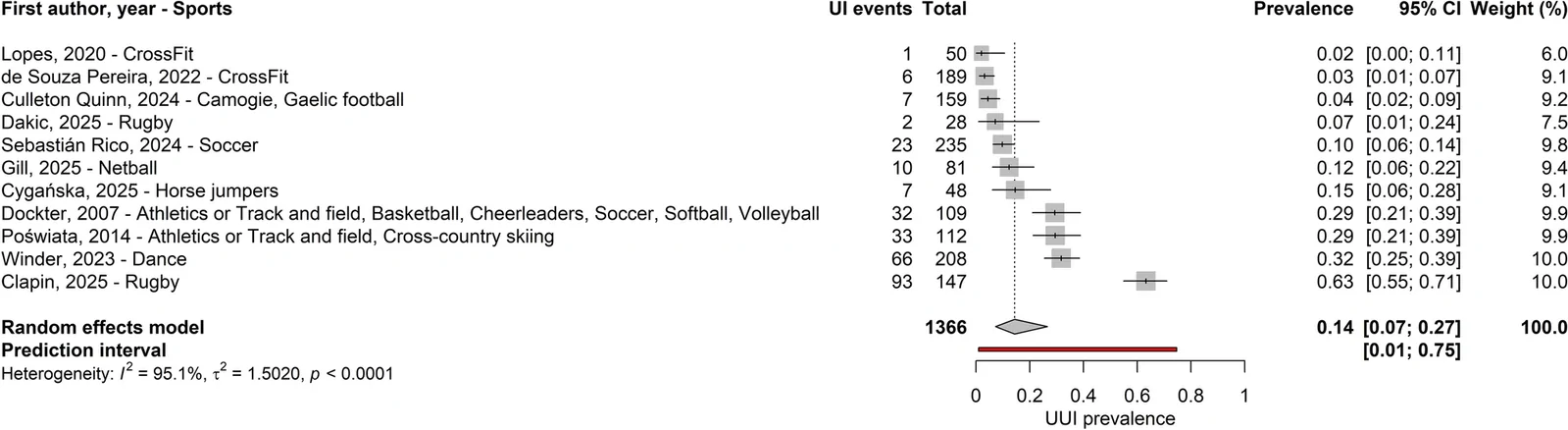
Forest plot of the pooled prevalence of urgency urinary incontinence (UUI) in women athletes aged 18–45 years. *CI* confidence interval

**Fig. 5:**
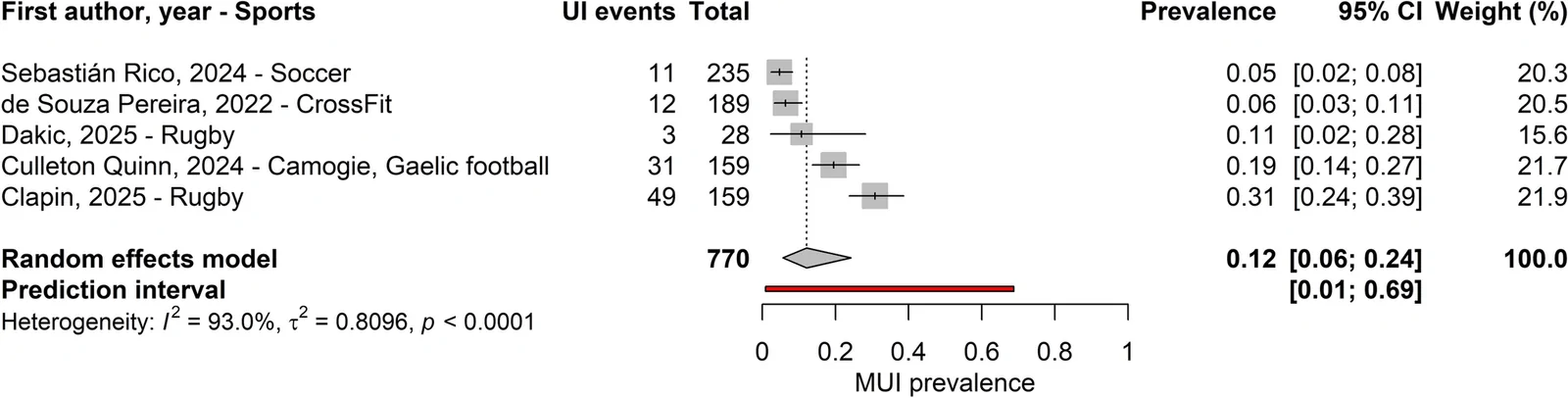
Forest plot of the pooled prevalence of mixed urinary incontinence (MUI) in women athletes aged 18–45 years. *CI* confidence interval, *UI* urinary incontinence

**Fig. 6:**
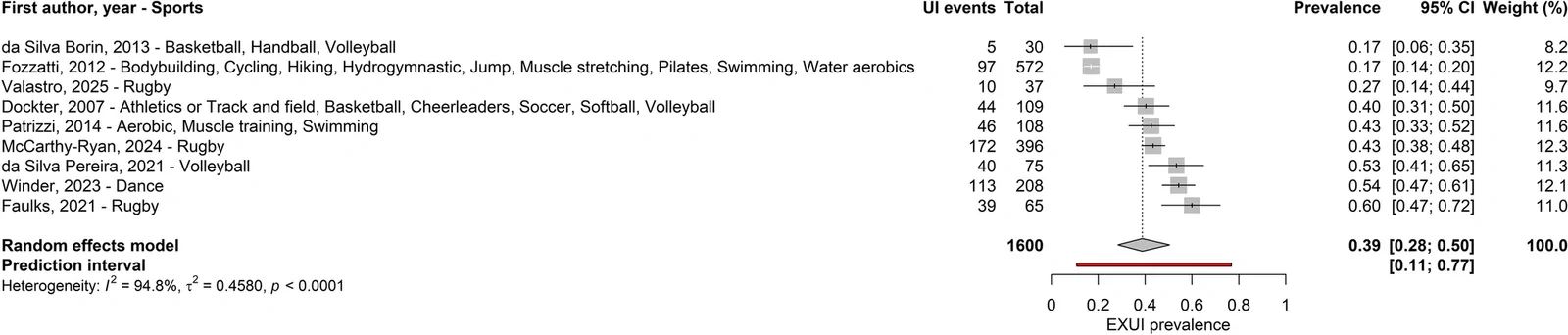
Forest plot of the pooled prevalence of exercise-related/exertional urinary incontinence (EXUI) in women athletes aged 18–45 years. *CI* confidence interval, *UI* urinary incontinence

Overall pooled UI prevalence in women athletes was 39% (95% CI 33–46; Fig. 2), with substantial heterogeneity (*I*^2^ = 94.3%). Pooled prevalences by UI subtype were 33% for SUI (95% CI 27–41; Fig. 3), 14% for UUI (95% CI 7–27; Fig. 4), 12% for MUI (95% CI 6–24; Fig. 5) and 39% for EXUI (95% CI 28–50; Fig. 6), with similar substantial heterogeneity (*I*^2^ > 93%). Sensitivity analyses revealed slight changes when considering only nulliparous samples (UI: 34%, SUI: 33%, UUI: 16%, EXUI: 28%) and only validated screening tools (UI: 42%, SUI: 32%, UUI: 13%, EXUI: 38%). Unexplained heterogeneity persists despite methodological restrictions (*I*^2^ = 81–96%). No sport-label classification meaningfully reduced between-cohort heterogeneity in meta-regression analyses.

**Fig. 7:**
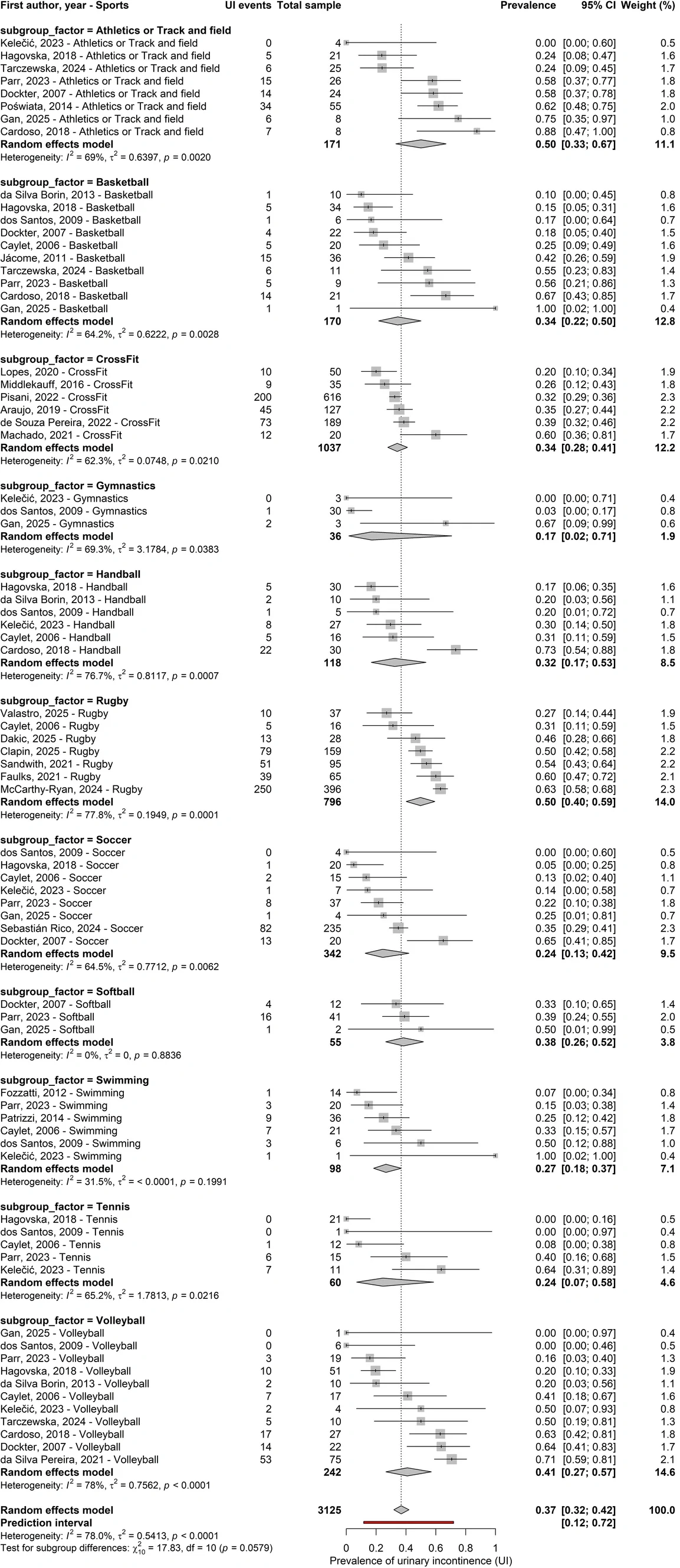
Forest plot of the pooled prevalence and subgroup differences of urinary incontinence (UI) in women athletes aged 18–45 years by sport discipline. *CI* confidence interval

Across sport disciplines, rugby showed the highest pooled prevalence of UI (50%, 95% CI 40–59), exceeding the overall estimate, whereas swimming exhibited the lowest (27%, 95% CI 18–37). However, differences between sport disciplines were statistically inconclusive in the meta-analysis (*p* = 0.058, *I*^2^ = 78%; Fig. 7) and not significant in the meta-regression (QM(10) = 11.71; *p* = 0.304; Table 3). This was consistent with sensitivity analyses in only nulliparous samples (QM(8) = 4.85, *p* = 0.773, *n* = 1606) and only validated screening tools (QM(8) = 9.18, *p* = 0.327, *n* = 2729).

**Table 3:** Mixed-effects meta-regression of the odds of urinary incontinence by sport discipline in women athletes aged 18–45 years

Comparison	Reference	OR (95% CI)	*p*-value	Comparison		Reference	
				*n* UI (total)	*k*	*n* UI (total)	*k*
Basketball	Athletics or track and field	0.53 (0.21–1.30)	0.164	57 (170)	10	87 (171)	8
CrossFit	Athletics or track and field	0.52 (0.21–1.29)	0.160	349 (1037)	6	87 (171)	8
CrossFit	Basketball	0.99 (0.41–2.37)	0.974	349 (1037)	6	57 (170)	10
Gymnastics	Athletics or track and field	0.19 (0.03–1.06)	0.058	3 (36)	3	87 (171)	8
Gymnastics	Basketball	0.35 (0.06–1.97)	0.235	3 (36)	3	57 (170)	10
Gymnastics	CrossFit	0.36 (0.06–2.01)	0.243	3 (36)	3	349 (1037)	6
Handball	Athletics or track and field	0.49 (0.18–1.34)	0.165	43 (118)	6	87 (171)	8
Handball	Basketball	0.93 (0.35–2.46)	0.886	43 (118)	6	57 (170)	10
Handball	CrossFit	0.94 (0.35–2.52)	0.910	43 (118)	6	349 (1037)	6
Handball	Gymnastics	2.65 (0.45–15.7)	0.283	43 (118)	6	3 (36)	3
Rugby	Athletics or track and field	0.94 (0.39–2.26)	0.881	447 (796)	7	87 (171)	8
Rugby	Basketball	1.77 (0.76–4.13)	0.187	447 (796)	7	57 (170)	10
Rugby	CrossFit	1.80 (0.76–4.24)	0.181	447 (796)	7	349 (1037)	6
Rugby	Gymnastics	5.03 (0.91–27.9)	0.065	447 (796)	7	3 (36)	3
Rugby	Handball	1.90 (0.73–4.94)	0.187	447 (796)	7	43 (118)	6
Soccer	Athletics or track and field	0.34 (0.13–0.90)	0.030	108 (342)	8	87 (171)	8
Soccer	Basketball	0.65 (0.25–1.66)	0.364	108 (342)	8	57 (170)	10
Soccer	CrossFit	0.66 (0.25–1.70)	0.386	108 (342)	8	349 (1037)	6
Soccer	Gymnastics	1.84 (0.32–10.7)	0.497	108 (342)	8	3 (36)	3
Soccer	Handball	0.70 (0.25–1.96)	0.492	108 (342)	8	43 (118)	6
Soccer	Rugby	0.37 (0.15–0.92)	0.032	108 (342)	8	447 (796)	7
Softball	Athletics or track and field	0.62 (0.17–2.27)	0.471	21 (55)	3	87 (171)	8
Softball	Basketball	1.17 (0.33–4.21)	0.805	21 (55)	3	57 (170)	10
Softball	CrossFit	1.19 (0.33–4.30)	0.789	21 (55)	3	349 (1037)	6
Softball	Gymnastics	3.34 (0.47–23.7)	0.228	21 (55)	3	3 (36)	3
Softball	Handball	1.26 (0.33–4.86)	0.737	21 (55)	3	43 (118)	6
Softball	Rugby	0.66 (0.19–2.35)	0.524	21 (55)	3	447 (796)	7
Softball	Soccer	1.81 (0.48–6.83)	0.379	21 (55)	3	108 (342)	8
Swimming	Athletics or track and field	0.37 (0.13–1.05)	0.063	24 (98)	6	87 (171)	8
Swimming	Basketball	0.69 (0.25–1.94)	0.488	24 (98)	6	57 (170)	10
Swimming	CrossFit	0.71 (0.25–1.99)	0.509	24 (98)	6	349 (1037)	6
Swimming	Gymnastics	1.97 (0.32–12.1)	0.461	24 (98)	6	3 (36)	3
Swimming	Handball	0.75 (0.24–2.28)	0.608	24 (98)	6	43 (118)	6
Swimming	Rugby	0.39 (0.14–1.08)	0.070	24 (98)	6	447 (796)	7
Swimming	Soccer	1.07 (0.36–3.19)	0.899	24 (98)	6	108 (342)	8
Swimming	Softball	0.59 (0.15–2.38)	0.460	24 (98)	6	21 (55)	3
Tennis	Athletics or track and field	0.42 (0.12–1.41)	0.160	14 (60)	5	87 (171)	8
Tennis	Basketball	0.79 (0.24–2.61)	0.698	14 (60)	5	57 (170)	10
Tennis	CrossFit	0.80 (0.24–2.67)	0.718	14 (60)	5	349 (1037)	6
Tennis	Gymnastics	2.24 (0.33–15.1)	0.408	14 (60)	5	3 (36)	3
Tennis	Handball	0.85 (0.24–3.04)	0.799	14 (60)	5	43 (118)	6
Tennis	Rugby	0.45 (0.14–1.46)	0.181	14 (60)	5	447 (796)	7
Tennis	Soccer	1.22 (0.35–4.26)	0.757	14 (60)	5	108 (342)	8
Tennis	Softball	0.67 (0.15–3.08)	0.608	14 (60)	5	21 (55)	3
Tennis	Swimming	1.14 (0.30–4.25)	0.851	14 (60)	5	24 (98)	6
Volleyball	Athletics or track and field	0.72 (0.30–1.71)	0.452	113 (242)	11	87 (171)	8
Volleyball	Basketball	1.35 (0.58–3.14)	0.479	113 (242)	11	57 (170)	10
Volleyball	CrossFit	1.37 (0.59–3.22)	0.465	113 (242)	11	349 (1037)	6
Volleyball	Gymnastics	3.85 (0.70–21.2)	0.122	113 (242)	11	3 (36)	3
Volleyball	Handball	1.45 (0.56–3.75)	0.439	113 (242)	11	43 (118)	6
Volleyball	Rugby	0.76 (0.34–1.74)	0.522	113 (242)	11	447 (796)	7
Volleyball	Soccer	2.09 (0.84–5.22)	0.114	113 (242)	11	108 (342)	8
Volleyball	Softball	1.15 (0.33–4.06)	0.824	113 (242)	11	21 (55)	3
Volleyball	Swimming	1.95 (0.71–5.33)	0.193	113 (242)	11	24 (98)	6
Volleyball	Tennis	1.72 (0.53–5.58)	0.369	113 (242)	11	14 (60)	5

Mixed-effects meta-regression of logit-transformed prevalence with random effects for between-study variance (estimator matched to forest analyses). Reciprocal contrasts were removed to avoid redundancy

*CI* confidence interval, *k* number of cohorts, *n* athletes contributing to each side of the contrast, *OR* odds ratio, *Ref* reference category, *UI* athletes with urinary incontinence

**Fig. 8:**
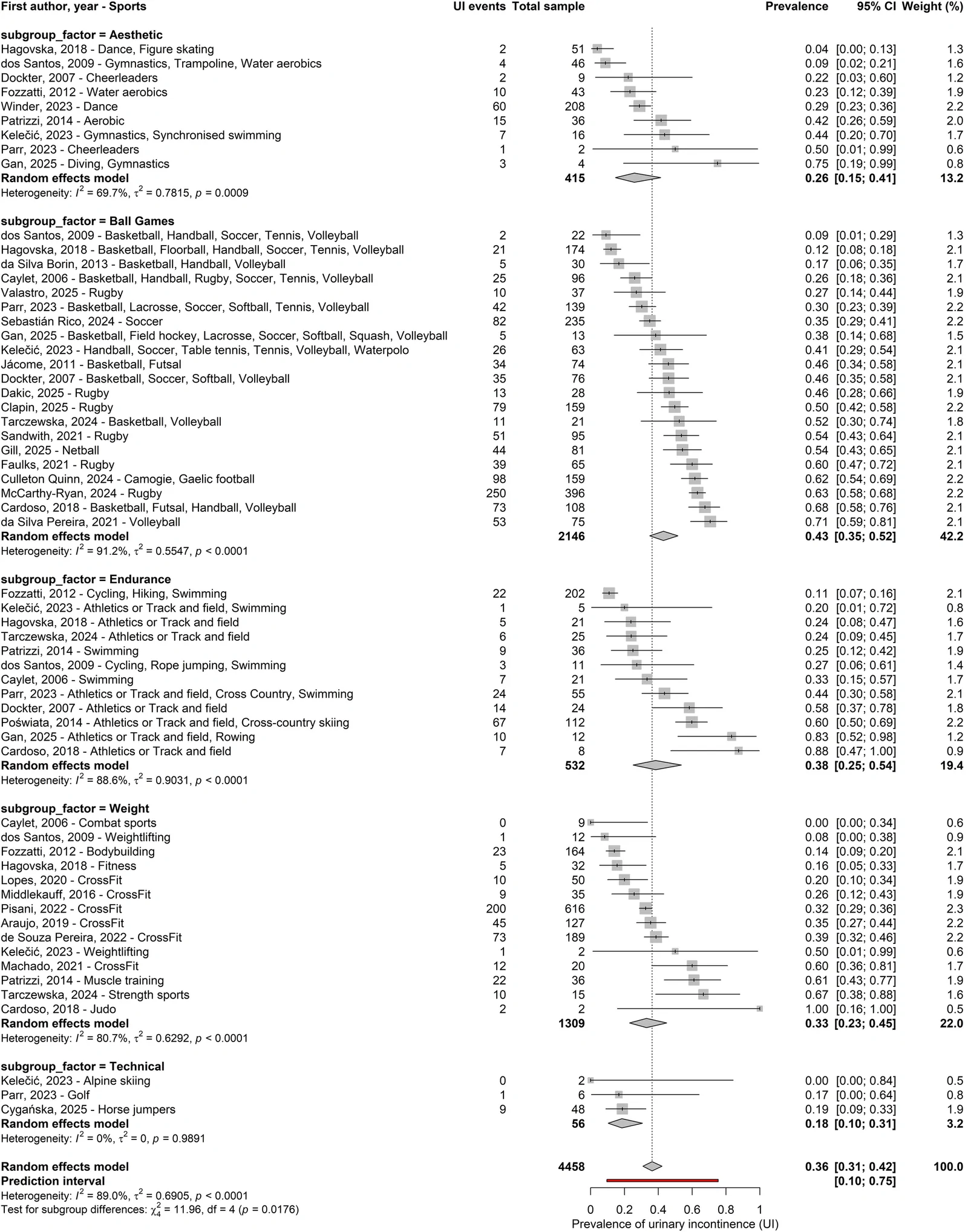
Forest plot of the pooled prevalence and subgroup differences of urinary incontinence (UI) in women athletes aged 18–45 years by sport modality. *CI* confidence interval

Across sport modalities, ball games showed the highest pooled prevalence of UI (43%, 95% CI 35–52), whereas technical sports reported the lowest (18%, 95% CI 10–31). Meta-analysis yielded significant differences (*p* = 0.018, *n* = 4,458; Fig. 8) but under a high heterogeneity (between-group *I*^2^ = 89.0%; within-group *I*^2^ range = 0–91.2%). Meta-regression did not identify sport modality as a significant moderator (QM(4) = 6.76; *p* = 0.149; Table 4). This was consistent with sensitivity analyses in only nulliparous samples (QM(4) = 2.81, *p* = 0.590, *n* = 2602) and only validated screening tools (QM(4) = 4.64, *p* = 0.326, *n* = 3902).

**Table 4:** Mixed-effects meta-regression of the odds of urinary incontinence by sport modality in women athletes aged 18–45 years

Comparison	Reference	OR (95% CI)	*p*-value	Comparison		Reference	
				*n* UI (total)	*k*	*n* UI (total)	*k*
Ball games	Aesthetic	2.16 (1.02–4.59)	**0.044**	998 (2146)	21	104 (415)	9
Endurance	Aesthetic	1.76 (0.75–4.11)	0.195	175 (532)	12	104 (415)	9
Endurance	Ball games	0.81 (0.42–1.56)	0.531	175 (532)	12	998 (2146)	21
Technical	Aesthetic	0.63 (0.14–2.77)	0.539	10 (56)	3	104 (415)	9
Technical	Ball games	0.29 (0.07–1.15)	0.079	10 (56)	3	998 (2146)	21
Technical	Endurance	0.36 (0.08–1.50)	0.161	10 (56)	3	175 (532)	12
Weight	Aesthetic	1.42 (0.62–3.26)	0.406	413 (1309)	14	104 (415)	9
Weight	Ball games	0.66 (0.35–1.23)	0.187	413 (1309)	14	998 (2146)	21
Weight	Endurance	0.81 (0.39–1.70)	0.576	413 (1309)	14	175 (532)	12
Weight	Technical	2.26 (0.54–9.42)	0.261	413 (1309)	14	10 (56)	3

Mixed-effects meta-regression of logit-transformed prevalence with random effects for between-study variance (estimator matched to forest analyses). Bold indicates *p* < 0.05. Reciprocal contrasts were removed to avoid redundancy

*CI* confidence interval, *k* number of cohorts, *n* athletes contributing to each side of the contrast, *OR* odds ratio, *Ref* reference category, *UI* athletes with urinary incontinence

**Fig. 9:**
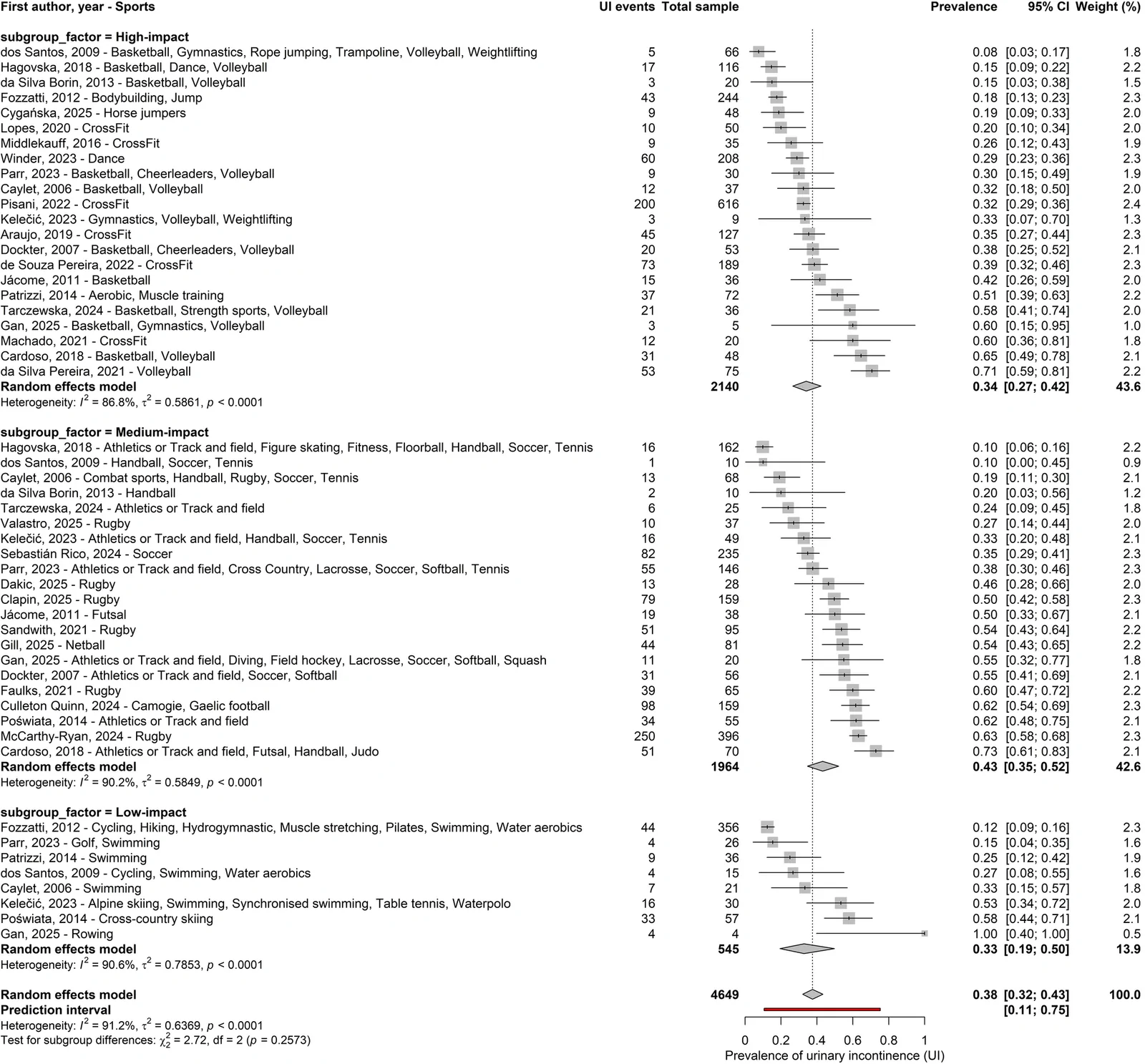
Forest plot of the pooled prevalence and subgroup differences of urinary incontinence (UI) in women athletes aged 18–45 years by sport impact. *CI* confidence interval

Across sports, medium-impact disciplines showed a higher pooled prevalence of UI (43%, 95% CI 35–52) than high-impact (34%, 95% CI 27–42) and low-impact (33%, 95% CI 19–50). However, sport impact showed no significant effect on UI pooled prevalences both in the meta-analysis (*p* = 0.257, *n* = 4649, *I*^2^ = 91.2%; Fig. 9) and meta-regression (QM(2) = 2.75; *p* = 0.253; Table 5). This was consistent with sensitivity analyses in only nulliparous samples (QM(2) = 1.83, *p* = 0.400, *n* = 2793) and only validated screening tools (QM(2) = 1.69, *p* = 0.428, *n* = 4093).

**Table 5:** Mixed-effects meta-regression of the odds of urinary incontinence by sport impact in women athletes aged 18–45 years

Comparison	Reference	OR (95% CI)	*p*-value	Comparison		Reference	
				*n* UI (total)	*k*	*n* UI (total)	*k*
Low impact	High impact	0.93 (0.45–1.95)	0.857	690 (2140)	22	121 (545)	8
Medium impact	High impact	1.47 (0.88–2.46)	0.137	690 (2140)	22	921 (1964)	21
Medium impact	Low impact	1.58 (0.76–3.29)	0.225	121 (545)	8	921 (1964)	21

Mixed-effects meta-regression of logit-transformed prevalence with random effects for between-study variance (estimator matched to forest analyses). Reciprocal contrasts were removed to avoid redundancy

*CI* confidence interval, *k* number of cohorts, *n* athletes contributing to each side of the contrast, *OR* odds ratio, *Ref* reference category, *UI* athletes with urinary incontinence

**Fig. 10:**
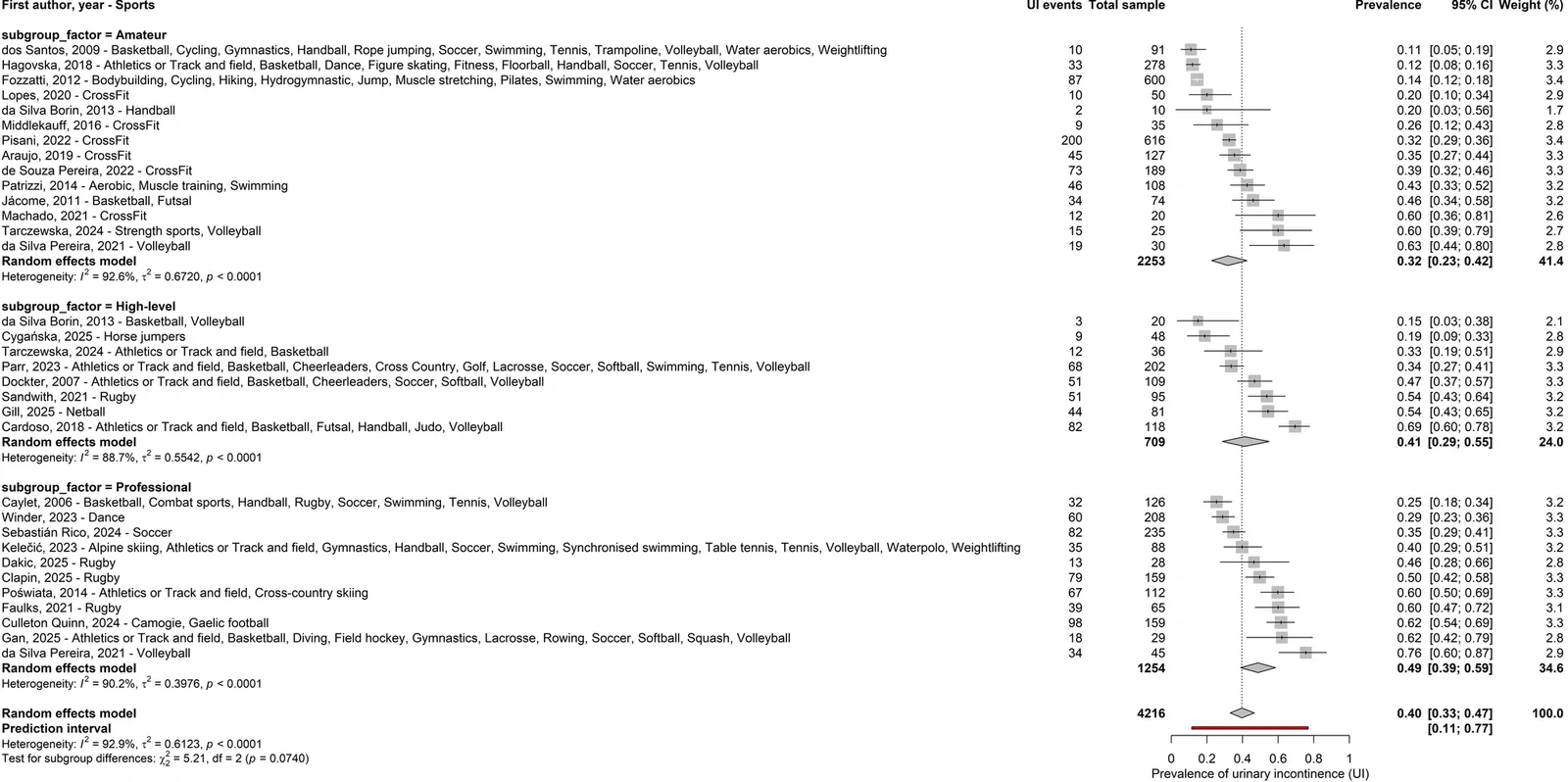
Forest plot of the pooled prevalence and subgroup differences of urinary incontinence (UI) in women athletes aged 18–45 years by competitive level. *CI* confidence interval

Across competitive levels, professional athletes showed the highest pooled prevalence of UI (49%, 95% CI 39–59), exceeding the overall estimate, whereas amateurs exhibited the lowest (32%, 95% CI 23–42). Although the subgroup meta-analysis did not reach conventional statistical significance in the meta-analysis (*p* = 0.074), the observed direction and magnitude of the estimates suggest a potential gradient across competitive levels, which could not be confirmed given substantial between-study heterogeneity (*I*^2^ = 92.9%). This trend was confirmed in meta-regression analysis (QM(2) = 5.19, *p* = 0.075; Table 6), where pairwise contrasts suggested higher odds of UI in professional athletes compared with amateur athletes. This pattern was not observed in analyses restricted to nulliparous samples (QM(2) = 2.69, *p* = 0.260; *n* = 2756), but approached statistical significance when restricted to studies using validated screening tools (QM(2) = 6.10, *p* = 0.050; *n* = 3660) (Fig. 10).

**Table 6:** Mixed-effects meta-regression of the odds of urinary incontinence by competitive level in women athletes aged 18–45 years

Comparison	Reference	OR (95% CI)	*p*-value	Comparison		Reference	
				*n* UI (total)	*k*	*n* UI (total)	*k*
High level	Amateur	1.51 (0.75–3.02)	0.246	595 (2253)	14	320 (709)	8
Professional	Amateur	2.05 (1.10–3.83)	0.024	595 (2253)	14	557 (1254)	11
Professional	High level	1.36 (0.66–2.80)	0.399	320 (709)	8	557 (1254)	11

Mixed-effects meta-regression of logit-transformed prevalence with random effects for between-study variance (estimator matched to forest analyses). Bold indicates *p* < 0.05. Reciprocal contrasts were removed to avoid redundancy

*CI* confidence interval, *k* number of cohorts, *N* athletes contributing to each side of the contrast, *OR* odds ratio, *Ref* reference category, *UI* athletes with urinary incontinence

**Fig. 11:**
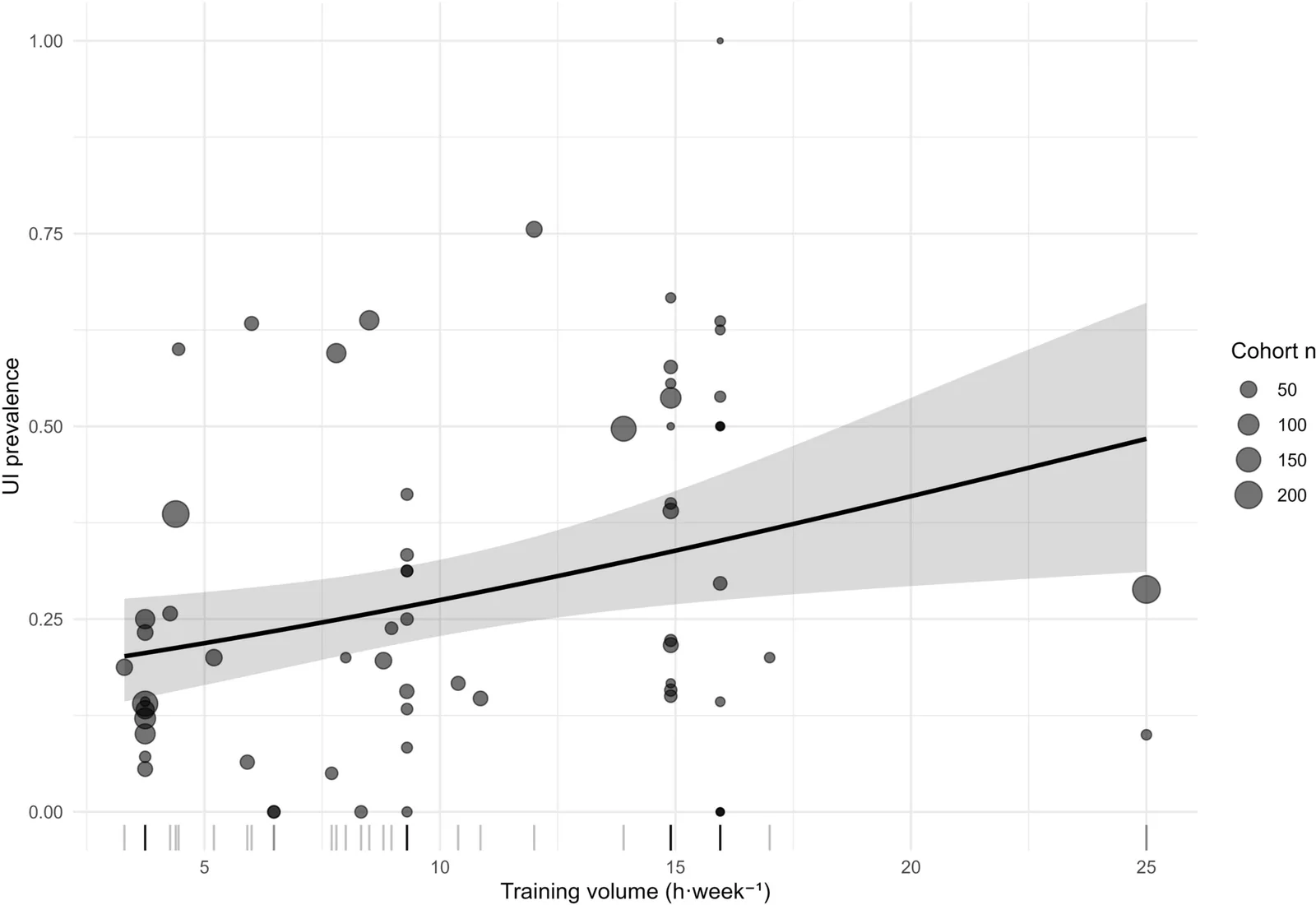
Association between weekly training volume (h·week⁻^1^) and urinary incontinence (UI) prevalence in women athletes aged 18–45 years. Each *point* represents an individual cohort, plotted as the observed UI prevalence (UI cases/total sample). Point size is proportional to cohort sample size (*n*). The *solid line* shows the predicted UI prevalence from a random-effects meta-regression (logit-transformed proportions; random-effects model estimator), and the *shaded band* represents the 95% confidence interval around the model prediction. *Rug marks along the x-axis* indicate the distribution of cohort training volumes

Meta-regression of training volume and intensity showed that greater weekly training hours were associated with higher odds of UI (per + 1 h·week⁻^1^: OR = 1.06, 95% CI 1.01–1.11, *p* = 0.011, *n* = 2362 athletes, Fig. 11). Using the sample SD, increments of 5.24 h·week⁻^1^ correspond to OR = 1.38 (95% CI 1.07–1.76). There was no evidence that the association differed by sports impact (*p* = 0.454) or competitive level (*p* = 0.750). This was consistent with sensitivity analyses in only nulliparous samples (per + 1 h·week⁻^1^: OR = 1.06, 95% CI 1.01–1.11; sample SD = 5.5 h·week⁻^1^, OR = 1.49, 95% CI 1.12–1.99, *p* = 0.011, *n* = 1308) and only validated screening tools (per + 1 h·week⁻^1^: OR = 1.08, 95% CI 1.02–1.13; sample SD = 5.56 h·week⁻^1^; OR = 1.40, 95% CI 1.05–1.85, *p* = 0.007, *n* = 2188).

## Discussion

This umbrella review and meta-analysis synthesised evidence from 14 systematic reviews and 32 primary studies to examine UI prevalence in adult women athletes and explore sport-related factors contributing to between-cohort variability. The findings indicate that UI is common in this population (39% overall), but characterised by substantial heterogeneity across cohorts. At the population level, commonly used sport-label classifications such as discipline, modality or impact category did not consistently explain differences in prevalence. Although some subgroups showed directional differences (e.g. higher point estimates in rugby and among professionals), these patterns were not robust moderators in meta-regression. In contrast, weekly training volume emerged as the most consistently reported exposure-based correlate of UI odds, although this association should be interpreted cautiously within a predominantly cross-sectional evidence base. Notably, no sport-label classification meaningfully reduced between-cohort heterogeneity.

The interpretation of sport-related patterns in UI among women athletes is fundamentally constrained by the quality of the existing synthesis literature. In this umbrella review, most included systematic reviews and meta-analyses were rated as low or critically low confidence, largely owing to absent protocol registration, inadequate assessment of bias in primary studies and limited consideration of methodological limitations when interpreting pooled estimates. Consequently, prior syntheses have reported widely divergent prevalence estimates and drawn inferences from heterogeneous populations and inconsistent sport classifications. By integrating a structured overview of reviews with a de novo meta-analysis of primary cohorts, the present study helps address these limitations and provides a more coherent athlete-centred synthesis of sport-related correlates of UI.

The absence of consistent associations between sport-label classifications and UI prevalence should not be interpreted as evidence of biomechanical equivalence between sports. Rather, these findings highlight limitations in how sport-related exposure is currently operationalised in the literature. Broad classifications such as “high-impact” or “low-impact” are coarse proxies that may inadequately capture pelvic floor loading, which is likely influenced by multiple interacting factors including movement patterns, intensity, fatigue, technique, recovery and individual neuromuscular characteristics [6, 25]. Previous reviews have similarly cautioned against over-interpreting sport type as a surrogate for pelvic floor stress, noting wide variability in UI prevalence even within the same disciplines [17, 22, 23, 42]. Importantly, this variability persisted even among cohorts examining similar populations or using only validated screening tools, as demonstrated across primary and sensitivity analyses. This pattern suggests that heterogeneity reflects more than methodological noise alone and likely indicates exposure misclassification when broad sport labels are used as proxies for pelvic floor loading. Exposure misclassification is therefore likely and may obscure sport-specific mechanisms when analyses are conducted at the population level.

Training volume showed the strongest association with UI prevalence, with higher weekly training hours linked to increased odds of UI. Similar observations have been reported in both athletic and recreationally active populations, where cumulative exercise exposure has been associated with pelvic floor symptoms independent of sport type [77–79], particularly at a high competitive level [80]. Cumulative exposure time may capture repeated loading, fatigue accumulation or insufficient recovery. However, it should not be interpreted as a direct mechanistic proxy for pelvic floor load. Rather, it reflects that the available data did not allow granular characterisation of true training intensity or pelvic floor-specific loading.

From a load–injury perspective, training volume and mechanical impact represent interrelated components of overall exposure, with injury risk depending on both the magnitude of load (e.g. intensity) and its accumulation over time (e.g. frequency and volume) [26]. In the present evidence base, however, only two studies reported Metabolic Equivalent of Task (MET)-based intensity metrics [56, 74], limiting dose–intensity modelling. Moreover, several cohorts explicitly report regular cross-training [47, 52, 56, 58, 63], suggesting that the total workload may be underestimated when sport-specific exposure is considered in isolation. Together, these limitations restrict the extent to which observed volume effects can be attributed to exposure duration per se, cumulative fatigue, or interactions between training duration and intensity.

These constraints, alongside the limited explanatory value of nominal sport classifications or generic mechanical proxies, support a more exposure-informed conceptual approach to pelvic floor stress and UI risk in women athletes. These findings indicate that pelvic floor exposure is unlikely to be meaningfully represented by sport type alone and instead requires a multidimensional construct integrating training exposure, pelvic floor-specific function and symptom response, where feasible. Specifically, a conceptual “pelvic floor dose” framework should integrate (i) exercise exposure describing the amount and scheduling of sport participation (e.g. weekly training hours, session frequency, competition density) [6, 26]; (ii) load descriptors capturing how that exposure is experienced, including external load (e.g. jump counts, sprint distance, ground contacts) and internal load (e.g. heart rate-based load, perceived exertion or neuromuscular responses such as electromyography) [26, 82, 83]; (iii) pelvic floor-specific structure and function, including muscle capacity, fatigability and displacement of pelvic support structures (e.g. ultrasound, or intravaginal pressure-based measures) [84–87]; and (iv) individual symptom responses during and following sport participation (e.g. leakage during specific tasks, symptom frequency, severity or recovery patterns) [10, 11].

Although integration of pelvic floor-specific assessments within broader exposure frameworks currently remains largely confined to research settings, it is essential for moving beyond indirect proxies of load. From a clinical and applied perspective, exposure-aware screening approaches that combine cumulative training demands with pelvic floor-specific considerations and symptom profiling may therefore offer a more informative basis for identifying potentially unsafe exposure patterns than sport type-based risk stratification alone. The high prevalence of UI, together with its association with training volume, supports the incorporation of exposure-aware screening, pelvic health education and timely referral within athlete care pathways. The emerging PFD-SENTINEL screening consensus for female athletes and broader female-athlete health frameworks provide practical entry points for embedding pelvic health in sport medicine workflows [25, 35]. Programmes should address knowledge gaps and stigma that normalise leakage, particularly in elite contexts where symptoms may be under-reported [10, 11, 88]. Given the early onset and high prevalence of UI among nulliparous athletes, interventions should not wait for parity or ageing-related risk factors to emerge. Women’s pelvic health physiotherapists and sport professionals should work closely with sports organisations to embed routine pelvic health screening within standard medical evaluations, ensure access to pelvic health education and actively challenge the cultural normalisation of leakage in sport.

Within this framework, competitive level warrants consideration as a potential modifier of cumulative exposure rather than as an independent risk factor. Prior reviews seldom examined competitive level as a moderator and generally did not perform level-stratified meta-analyses. Cross-sport syntheses focused on sport/impact rather than level [18, 21, 27], with broader reviews mentioning elite athletes without formal quantitative evaluation [16]. In our data, although the competitive level did not reach conventional significance, a subgroup test and a directional pairwise contrast suggested higher odds of UI among professional athletes compared with amateur athletes. Given the substantial within-level heterogeneity and wide prediction intervals, this signal should be interpreted cautiously and considered hypothesis generating. A plausible explanation might be greater cumulative exposure over time at the professional level [10, 11, 88]. However, the present analyses did not identify differential associations between weekly training volume and UI across competitive levels. Prospective level-stratified studies with granular workload metrics are therefore needed to disentangle the contributions of competitive context, cumulative load and recovery to pelvic floor symptom risk. The presence of elevated prevalence estimates among nulliparous professional athletes reinforces that exposure-related risk operates independently of obstetric history.

Several limitations should be acknowledged. Most available data describe prevalence rather than incidence, limiting inference on symptom onset, progression and reversibility in relation to training exposure. Consequently, it remains unclear whether higher training exposure primarily contributes to symptom onset, prolonged persistence or recurrent episodes of UI in women athletes. Pelvic floor-specific exposure metrics were rarely reported, necessitating reliance on indirect proxies such as sport labels and training volume. In this context, sport classification itself represents an additional source of uncertainty; although we applied a classification commonly used in pelvic floor research, alternative systems for categorising sport modalities could produce different estimates. Finally, the restriction to adult women improves aetiological specificity but limits generalisability to adolescent athletes, who represent an important population for future dedicated syntheses. Notwithstanding these limitations, several methodological strengths warrant consideration. Unlike prior syntheses that pooled broad and heterogeneous populations, the present review focused on a biologically and clinically defined population of adult women athletes (aged 18–45 years), improving aetiological specificity. In addition, sport context (discipline and modality), competitive level and continuous training exposure were examined using consistent eligibility criteria, exposure definitions and statistical models. This approach provides a more athlete-specific synthesis of sport-related correlates of UI within the constraints of the available evidence.

Relative to our initial protocol, we introduced some refinements to enhance precision and clinical relevance: (1) scope narrowed to UI only; (2) no planned comparison with the general population/sedentary controls was undertaken because of incompatibility of parity/age and measurement across datasets; (3) we added a de novo meta-analysis of primary studies to the umbrella review because existing reviews overlapped and did not provide the necessary subgroup/exposure granularity; (4) we introduced continuous exposure meta-regressions beyond the protocol’s subgroup framework; and (5) although the protocol targeted nulliparous women aged 18–45 years, some cohorts included mixed parity, addressed via nulliparous-only sensitivity analyses rather than outright exclusion.

## Conclusions

Overall, UI and related pelvic floor symptoms appear common across athletic populations, including nulliparous women. However, when examined collectively at a population level and within the constraints of the available evidence, commonly used sport-label classifications did not meaningfully explain between-cohort variability in UI prevalence. Although some directional differences were observed (e.g. higher point estimates in rugby or among professional athletes), these patterns were not robust moderators once heterogeneity was accounted for.

In contrast, exposure-related variables were more consistently associated with UI prevalence in the available data, although these associations reflect study-level reporting rather than direct pelvic floor-specific load quantification. Competitive level showed a directional trend toward higher prevalence among professional athletes, likely reflecting greater cumulative exposure rather than sport type per se, although this association requires confirmation in adequately powered, level-stratified studies. Most notably, cumulative training exposure, operationalised as weekly training volume, emerged as the most consistently reported exposure correlate of UI odds and remained robust across sport categories, impact classifications and sensitivity analyses. This finding should be interpreted as descriptive and hypothesis generating, and in the context of limited reporting of training intensity and pelvic floor-specific mechanical load. Nevertheless, the consistently high prevalence of symptoms across sports and competitive levels supports a precautionary athlete-centred approach to pelvic health screening, independent of unresolved mechanistic uncertainties.

Taken together, these findings suggest that UI risk in women athletes is better understood through an exposure-based framework than through nominal sport labels. From a clinical and performance perspective, this supports shifting pelvic health screening and prevention strategies away from sport type-based assumptions and toward monitoring cumulative training exposure and symptom response across all sports and competitive levels. Future research should prioritise prospective designs that integrate training exposure with pelvic floor-specific function and symptom trajectories to inform load management strategies and help define safe pelvic floor exposure thresholds in women athletes.

## Supplementary Information

Below is the link to the electronic supplementary material.

Supplementary file1 (PDF 896 KB)

Supplementary file2 (R 39 KB)

Supplementary file3 (XLSX 19 KB)

## Declarations

### Availability of data and material

All data extracted and used for the analysis in this systematic review and meta-analysis are included in the supplementary material.

### Code availability

The R Code is included in the supplementary material.
