# Supplementary material for: Urinary Incontinence in Women Athletes: Umbrella Review and Meta-analysis of Sport-Related Factors
Source: Sports Med. 2026 Apr 16;56(9):2185–208. doi: 10.1007/s40279-026-02438-z (PMC13499830; doi:10.1007/s40279-026-02438-z)
Supplement: Supplementary file 1 [file 40279_2026_2438_MOESM1_ESM.pdf]

ELECTRONIC SUPPLEMENTARY MATERIAL

Title: Urinary incontinence in women athletes: umbrella review and meta-analysis of sport-related factors

Journal: Sports Medicine

Authors:

Nuria Domínguez-Pérez[1], Irene Sevilla-Arrabal[2], Beatriz Navarro Brazález[1,3], María Torres Lacomba[1,3] & Javier Courel-Ibáñez[2].

Affiliations:

[1]Physiotherapy in Women's Health Research Group, Nursing and Physiotherapy Department, Universidad de Alcalá, Alcalá de Henares, Madrid, Spain.

[2]Department of Physical Education and Sport, Faculty of Sport Sciences, University of Granada, Granada, Spain.

[3]Ramón y Cajal Institute of Health Research-IRYCIS, University Hospital of Ramón y Cajal, Madrid, Spain.

Corresponding author:

Javier Courel-Ibáñez, courel@ugr.es

APPENDIX 1: Search strategy

PubMed: 107 (29 September 2025)

#	Search terms	Results
1	"Urinary Incontinence"[MeSH] OR "Fecal Incontinence"[MeSH] OR "Pelvic Floor Disorders"[MeSH] OR "Pelvic Organ Prolapse"[MeSH] OR Diastasis[tiab]	61,841
2	"Pelvic Floor" OR "Genital Prolapse" OR "Anal Incontinence" OR "Faecal Incontinence"[tiab]	24,467
3	#1 OR #2	73,311
4	"Athletes"[MeSH] OR "Sports"[MeSH] OR "Exercise"[MeSH] OR "Women Athletes"[tiab] OR "Female Athletes"[tiab]	403,103
5	"Sedentary Behavior"[MeSH] OR "General Population"[tiab] OR "Non-Athletes"[tiab] OR "Inactive Women"[tiab] OR "Healthy Women"[tiab]	187,174
6	#4 OR #5	574,549
7	"Systematic Review"[pt] OR "Meta-Analysis"[pt]	400,000
8	"systematic review"[tiab] OR "meta-analysis"[tiab] OR "review of reviews"[tiab] OR "overview"[tiab]	771,187
9	#7 OR #8	813,191
10	#3 AND #6 AND #7	118
11	Refined by: Publication year >= 2010	107

Web of Science: 276 (29 September 2025)

#	Search terms	Results
1	TS=("Pelvic Floor" OR "Urinary Incontinence" OR "Fecal Incontinence" OR "Anal Incontinence" OR "Faecal Incontinence" OR "Pelvic Prolapse" OR "Genital Prolapse" OR "Pelvic Organ Prolapse" OR "Diastasis")	72,357
2	TS=("Athletes" OR "Sports" OR "Exercise" OR "Women Athletes" OR "Female Athletes" OR "Sedentary Behavior" OR "General Population" OR "Non-Athletes" OR "Inactive Women" OR "Healthy Women")	1,022,163
3	TS=("Systematic Review" OR "Meta-Analysis")	651,745
4	#1 AND #2 AND #3	367
5	Refined by: Publication year >= 2010	276

Scopus: 376 (29 September 2025)

#	Search terms	Results
1	TITLE-ABS-KEY("Pelvic Floor" OR "Urinary Incontinence" OR "Fecal Incontinence" OR "Anal Incontinence" OR "Pelvic Prolapse" OR "Genital Prolapse" OR "Pelvic Organ Prolapse" OR "Diastasis")	97,729
2	TITLE-ABS-KEY("Athletes" OR "Sports" OR "Exercise" OR "Women Athletes" OR "Female Athletes" OR "Sedentary Lifestyle" OR "General Population" OR "Non-Athletes" OR "Inactive Women" OR "Healthy Women")	1,479,471
3	TITLE-ABS-KEY("Systematic Review" OR "Meta-Analysis")	893,163
4	#1 AND #2 AND #3	711
5	Refined by: Publication year >= 2010, Review	376

Cochrane: 21 (29 September 2025)

#	Search terms	Results
1	("Pelvic Floor" OR "Urinary Incontinence" OR "Fecal Incontinence" OR "Anal Incontinence" OR "Faecal Incontinence" OR "Pelvic Prolapse" OR "Genital Prolapse" OR "Pelvic Organ Prolapse" OR "Diastasis")	120
2	("Athletes" OR "Sports" OR "Exercise" OR "Women Athletes" OR "Female Athletes" OR "Sedentary Behaviour" OR "General Population" OR "Non-Athletes" OR "Inactive Women" OR "Healthy Women")	1,108
3	("Systematic Review" OR "Meta-Analysis")	4,669

4	#1 AND #2 AND #3	23
5	Refined by: Publication year >= 2010	21

CINAHL: 37 (29 September 2025)

#	Search terms	Results
1	(MH "Pelvic Floor" OR MH "Pelvic Floor Disorders" OR MH "Urinary Incontinence" OR MH "Fecal Incontinence" OR MH "Pelvic Organ Prolapse" OR diastasis)	16,211
2	(MH "Athletes" OR MH "Sports" OR MH "Exercise" OR "women athletes" OR "female athletes" OR "sedentary lifestyle" OR "general population" OR "non-athletes" OR "inactive women" OR "healthy women")	240,792
3	(PT "Systematic Review" OR PT "Meta Analysis")	197,634
4	#1 AND #2 AND #3	42
5	Refined by: Publication year >= 2010	37

APPENDIX 2. Excluded reviews after full-text review with reasons

1. Balk EM, Rofeberg VN, Adam GP, Kimmel HJ, Trikalinos TA, Jeppson PC. Pharmacologic and Nonpharmacologic Treatments for Urinary Incontinence in Women: A Systematic Review and Network Meta-analysis of Clinical Outcomes. *Ann Intern Med*. 2019;170(7):465-479. doi:10.7326/M18-3227

Excluded: Treatment-focused; not athlete stratified.

2. Bascur-Castillo C, Carrasco-Portiño M, Valenzuela-Peters R, Orellana-Gaete L, Viveros-Allende V, Ruiz Cantero MT. Effect of conservative treatment of pelvic floor dysfunctions in women: An umbrella review. *Intl J Gynecology & Obste*. 2022;159(2):372-391. doi:10.1002/ijgo.14172

Excluded: Treatment effect, not prevalence; general population.

3. Bø K, Driusso P, Jorge CH. Can you breathe yourself to a better pelvic floor? A systematic review. *Neurourology and Urodynamics*. 2023;42(6):1261-1279. doi:10.1002/nau.25218

Excluded: Intervention focus; no UI prevalence data.

4. Bø K, Herbert RD. There is not yet strong evidence that exercise regimens other than pelvic floor muscle training can reduce stress urinary incontinence in women: a systematic review. *Journal of Physiotherapy*. 2013;59(3):159-168. doi:10.1016/S1836-9553(13)70180-2

Excluded: Treatment effects; general population.

5. Bø K, Hilde G. Does it work in the long term?—A systematic review on pelvic floor muscle training for female stress urinary incontinence. *Neurourology and Urodynamics*. 2013;32(3):215-223. doi:10.1002/nau.22292

Excluded: Treatment effect, not prevalence; general population.

6. Casey EK, Temme K. Pelvic floor muscle function and urinary incontinence in the female athlete. *The Physician and Sportsmedicine*. 2017;45(4):399-407. doi:10.1080/00913847.2017.1372677

Excluded: No systematic review.

7. Chisholm L, Delpe S, Priest T, Reynolds WS. Physical Activity and Stress Incontinence in Women. *Curr Bladder Dysfunct Rep*. 2019;14(3):174-179. doi:10.1007/s11884-019-00519-6

Excluded: No systematic review.

8. Dakic JG, Hay-Smith J, Cook J, Lin KY, Calo M, Frawley H. Effect of Pelvic Floor Symptoms on Women's Participation in Exercise: A Mixed-Methods Systematic Review With Meta-analysis. *Journal of Orthopaedic & Sports Physical Therapy*. 2021;51(7):345-361. doi:10.2519/jospt.2021.10200

Excluded: Not UI prevalence.

9. Eliks M, Zgorzalewicz-Stachowiak M, Zeńczak-Praga K. Application of Pilates-based exercises in the treatment of chronic non-specific low back pain: state of the art. *Postgraduate Medical Journal*. 2019;95(1119):41-45. doi:10.1136/postgradmedj-2018-135920

Excluded: No UI prevalence outcome, no UI focus

10. Fukuda FS, Arbieto ERM, Da Roza T, Luz SCT Da. Pelvic Floor Muscle Training in Women Practicing High-impact Sports: A Systematic Review. *Int J Sports Med*. 2022;44(6):397-405. doi:10.1055/a-1939-4798

Excluded: No UI prevalence outcome, no UI focus

11. Gan, Z.S., Smith, A.L. Urinary Incontinence in Elite Female Athletes. *Curr Urol Rep* 2023. 24, 51–58. doi: 10.1007/s11934-022-01133-6

Excluded: No systematic review.

12. Giagio S, Innocenti T, Pillastrini P, Gava G, Salvioli S. What is known from the existing literature about the available interventions for pelvic floor dysfunction among female athletes? A scoping review. *Neurourology and Urodynamics*. 2022;41(2):573-584. doi:10.1002/nau.24883

Excluded: Scoping review; no UI prevalence data.

13. Hay-Smith EJC, Herderschee R, Dumoulin C, Herbison GP. Comparisons of approaches to pelvic floor muscle training for urinary incontinence in women. Cochrane Incontinence Group, ed. *Cochrane Database of Systematic Reviews*. Published online December 7, 2011. doi:10.1002/14651858.CD009508

Excluded: Treatment effect only; not UI prevalence; general adult population, not athlete-specific

14. Heath A, Folan S, Ripa B, et al. Stress Urinary Incontinence in Female Athletes. *Journal of Women's Health Physical Therapy*. 2014;38(3):104-109. doi:10.1097/JWH.0000000000000016

Excluded: No systematic review.

15. Imamura M, Williams K, Wells M, McGrother C. Lifestyle interventions for the treatment of urinary incontinence in adults. Cochrane Incontinence Group, ed. *Cochrane Database of Systematic Reviews*. 2015;2015(12). doi:10.1002/14651858.CD003505.pub5

Excluded: Treatment-focused; adult women but not stratified by sport or prevalence.

16. Jacomo RH, Nascimento TR, Lucena Da Siva M, et al. Exercise regimens other than pelvic floor muscle training cannot increase pelvic muscle strength-a systematic review. *Journal of Bodywork and Movement Therapies*. 2020;24(4):568-574. doi:10.1016/j.jbmt.2020.08.005

Excluded: Focus on strength outcomes, not prevalence or athletic groups.

17. Kannan P, Winser S, Goonetilleke R, Cheing G. Ankle positions potentially facilitating greater maximal contraction of pelvic floor muscles: a systematic review and meta-analysis. *Disability and Rehabilitation*. 2019;41(21):2483-2491. doi:10.1080/09638288.2018.1468934

Excluded: Not focused on UI prevalence or athletic population;

18. Kneißler E, Zentgraf K. Prevalence of urinary incontinence in female gymnasts: a systematic review. *Ger J Exerc Sport Res*. Published online 2024. doi:10.1007/s12662-024-01003-y

Excluded: No studies on the target population.

19. Lemos AQ, Brasil CA, Valverde D, Ferreira JDS, Lordêlo P, Sá KN. The pilates method in the function of pelvic floor muscles: Systematic review and meta-analysis. *Journal of Bodywork and Movement Therapies*. 2019;23(2):270-277. doi:10.1016/j.jbmt.2018.07.002

Excluded: Muscle function change, not UI prevalence or sport-specific groups.

20. Magor J, Martin R, Bird ML. Athletes' Knowledge of Pelvic Floor Dysfunction and Their Knowledge of and Engagement with Pelvic Floor Muscle Training: A Scoping Review. *IJERPH*. 2025;22(1):104. doi:10.3390/ijerph22010104

Excluded: No UI prevalence data.

21. Marín Mora CM, Fonseca Chaves M. Prevalence and risk factors of stress urinary incontinence in nulliparous female athletes. *PensarMov*. 2019;17(2):e35033. doi:10.15517/pensarmov.v17i2.35033

Excluded: No systematic review.

22. McClurg D, Pollock A, Campbell P, et al. Conservative interventions for urinary incontinence in women: an Overview of Cochrane systematic reviews. Cochrane Incontinence Group, ed. *Cochrane Database of Systematic Reviews*. Published online September 1, 2016. doi:10.1002/14651858.CD012337

Excluded: Treatment-focused umbrella review, no prevalence analysis or athlete subgroup.

23. Menezes EC, Da Silva Pereira F, Porto RM, Fank F, Mazo GZ. Effect of exercise on female pelvic floor morphology and muscle function: a systematic review. *Int Urogynecol J*. 2023;34(5):963-977. doi:10.1007/s00192-022-05375-3

Excluded: Treatment umbrella review; no meta-analysis of prevalence.

24. Moroni R, Magnani P, Haddad J, Castro R, Brito L. Conservative Treatment of Stress Urinary Incontinence: A Systematic Review with Meta-analysis of Randomized Controlled Trials. *Rev Bras Ginecol Obstet*. 2016;38(02):097-111. doi:10.1055/s-0035-1571252

Excluded: Treatment-focused; no athlete-specific sample or prevalence estimates.

25. Rebullido TR, Gómez-Tomás C, Faigenbaum AD, Chulvi-Medrano I. The prevalence of urinary incontinence among adolescent female athletes: A systematic review. *J Funct Morphol Kinesiol*. 2021;6(1). doi:10.3390/jfmk6010012

Excluded: No studies on the target population.

26. Robertson B, Harding KE. Outcomes With Individual Versus Group Physical Therapy for Treating Urinary Incontinence and Low Back Pain: A Systematic Review and Meta-Analysis of Randomized Controlled Trials. *Archives of Physical Medicine and Rehabilitation*. 2014;95(11):2187-2198. doi:10.1016/j.apmr.2014.07.005

Excluded: Intervention-focused; not UI prevalence or sport/exercise population.

27. Romero-Franco N, Molina-Mula J, Bosch-Donate E, Casado A. Therapeutic exercise to improve pelvic floor muscle function in a female sporting population: a systematic review and meta-analysis. *Physiotherapy*. 2021;113:44-52. doi:10.1016/j.physio.2021.04.006

Excluded: Treatment-focused; no UI prevalence.

28. Thomaz RP, Colla C, Darski C, Paiva LL. Influence of pelvic floor muscle fatigue on stress urinary incontinence: a systematic review. *Int Urogynecol J*. 2018;29(2):197-204. doi:10.1007/s00192-017-3538-6

Excluded: Fatigue focus, not UI prevalence for sport-specific groups.

29. Tung MJ-Y, Lantz GA, Lopes AD, *et al*. Injuries in weightlifting and powerlifting: an updated systematic review. *BMJ Open Sport & Exercise Medicine.* 2024;10:e001884. doi:10.1136/bmjsem-2023-001884

Excluded: Injury focus, not UI prevalence for sport-specific groups.

30. Villa Del Pino I, Chillón Martínez R. La adherencia al entrenamiento muscular del suelo pélvico en las mujeres con incontinencia urinaria. Revisión sistemática de la literatura. *Fisioterapia*. 2019;41(4):227-236. doi:10.1016/j.ft.2019.03.005

Excluded: Adherence study; no prevalence estimates or athlete-specific data.

31. Villa-Del-Pino I, Jiménez-Rejano JJ, Rebollo-Salas M, Rodríguez-Domínguez ÁJ, Suárez-Serrano CM. Compliance and Adherence to Pelvic Floor Exercise Therapy in People with Pelvic Floor Disorders: A Systematic Review and Meta-Analysis. *Life*. 2025;15(4):613. doi:10.3390/life15040613

Excluded: Focus on adherence/compliance; no prevalence or athlete subgroup.

32. Zhao L, Li N, Gu L, et al. Attitudes and Perceptions of eHealth Among Female Patients with Urinary Incontinence and Healthcare Providers: A Systematic Review and Thematic Synthesis. *RMHP*. 2025;Volume 18:785-800. doi:10.2147/RMHP.S496997

Excluded: No UI prevalence or athlete-focused review.

APPENDIX 3. AMSTAR 2 quality assessment of included systematic reviews.

First author, year	# 1	# 2	# 3	# 4	# 5	# 6	# 7	# 8	# 9	# 10	# 11	# 12	# 13	# 14	# 15	# 16	Global rating
Almousa, 2019	Y	N	Y	Y	Y	Y	N	Y	Y	P	NA	NA	P	NA	N	Y	Critically low
Alvarez-Garcia, 2022	Y	Y	Y	Y	Y	Y	N	Y	Y	N	N	Y	Y	Y	Y	Y	Critically low
Castro, 2025	Y	Y	Y	Y	Y	Y	N	Y	Y	N	NA	NA	Y	NA	NA	Y	Low
Cerruto, 2020	Y	N	Y	Y	Y	Y	N	Y	P	N	Y	Y	Y	Y	Y	Y	Critically low
Culleton-Quinn, 2022	Y	N	Y	Y	Y	Y	N	Y	N	N	NA	N	N	P	N	Y	Critically low
De Mattos Lourenco, 2018	Y	N	P	Y	P	P	N	Y	N	N	N	N	N	P	N	Y	Critically low
Dominguez-Antuña, 2023	Y	N	P	Y	Y	Y	N	Y	P	N	Y	N	N	P	N	Y	Critically low
Garcia-Perdomo, 2022	Y	N	Y	Y	Y	Y	N	Y	Y	N	Y	Y	Y	Y	N	Y	Critically low
Martins, 2017	Y	N	Y	Y	Y	Y	N	Y	N	N	NA	NA	Y	NA	NA	Y	Critically low
Pires, 2020	Y	Y	Y	Y	Y	Y	N	Y	Y	N	Y	Y	Y	Y	Y	Y	Low
Romero Parra, 2024	Y	N	Y	Y	Y	Y	N	Y	Y	N	NA	NA	Y	NA	N	Y	Critically low
Silva Pereira, 2017	Y	Y	Y	Y	Y	Y	N	Y	N	N	NA	NA	Y	NA	NA	Y	Critically low
Syeda, 2024	Y	N	Y	Y	Y	Y	N	Y	Y	N	NA	NA	Y	NA	NA	Y	Critically low
Teixeira, 2018	Y	N	Y	Y	Y	Y	N	Y	Y	N	NA	NA	Y	P	N	Y	Critically low

Notes:
Item 1. Did the research questions and inclusion criteria for the review include the components of PICO? For Yes: □ Population □ Intervention □ Comparator group □ Outcome Optional (recommended) □ Timeframe for follow-up
Item 2. Did the report of the review contain an explicit statement that the review methods were established prior to the conduct of the review and did the report justify any significant deviations from the protocol? For Partial Yes: The authors state that they had a written protocol or guide that included ALL the following: □ review question(s) □ a search strategy □ inclusion/exclusion criteria □ a risk of bias assessment For Yes: As for partial yes, plus the protocol should be registered and should also have specified: □ a meta-analysis/synthesis plan, if appropriate, and □ a plan for investigating causes of heterogeneity □ justification for any deviations from the protocol
Item 3. Did the review authors explain their selection of the study designs for inclusion in the review? For Yes, the review should satisfy ONE of the following: □ Explanation for including only RCTs □ OR Explanation for including only observational designs □ OR Explanation for including both
Item 4. Did the review authors use a comprehensive literature search strategy? For Partial Yes (all the following): □ searched at least 2 databases (relevant to research question) □ provided key word and/or search strategy □ justified publication restrictions (e.g. language) For Yes, should also have (all the following): □ searched the reference lists / bibliographies of included studies □ searched trial/study registries □ included/consulted content experts in the field □ where relevant, searched for grey literature □ conducted search within 24 months of completion of the review
Item 5. Did the review authors perform study selection in duplicate? For Yes, either ONE of the following: □ at least two reviewers independently agreed on selection of eligible studies and achieved consensus on which studies to include □ OR two reviewers selected a sample of eligible studies and achieved good agreement (at least 80 percent), with the remainder selected by one reviewer.
Item 6. Did the review authors perform data extraction in duplicate? For Yes, either ONE of the following: □ at least two reviewers achieved consensus on which data to extract from included studies □ OR two reviewers extracted data from a sample of eligible studies and achieved good agreement (at least 80 percent), with the remainder extracted by one reviewer.
Item 7. Did the review authors provide a list of excluded studies and justify the exclusions? For Partial Yes: □ provided list of exclusions with number of studies in the PRISMA or in-text. For Yes, must also have: □ Justified the exclusion from the review of each potentially relevant study
Item 8. Did the review authors describe the included studies in adequate detail? For Partial Yes (ALL the following): □ described populations □ described

interventions described comparators described outcomes described research designs For Yes, should also have ALL the following: described population in detail described intervention in detail (including doses where relevant) described comparator in detail (including doses where relevant) described study's setting timeframe for follow-up

Item 9. Did the review authors use a satisfactory technique for assessing the risk of bias (RoB) in individual studies that were included in the review? For Partial Yes, must have assessed RoB: from confounding, and from selection bias For Yes, must also have assessed RoB: methods used to ascertain exposures and outcomes, and selection of the reported result from among multiple measurements or analyses of a specified outcome

Item 10. Did the review authors report on the sources of funding for the studies included in the review? For Yes Must have reported on the sources of funding for individual studies included in the review. Note: Reporting that the reviewers looked for this information but it was not reported by study authors also qualifies

Item 11. If meta-analysis was performed did the review authors use appropriate methods for statistical combination of results? For Yes: The authors justified combining the data in a meta-analysis AND they used an appropriate weighted technique to combine study results, adjusting for heterogeneity if present AND they statistically combined effect estimates from observational studies that were adjusted for confounding, rather than combining raw data, or justified combining raw data when adjusted effect estimates were not available AND they reported separate summary estimates for different study designs separately when two or more were included in the review

Item 12. If meta-analysis was performed, did the review authors assess the potential impact of RoB in individual studies on the results of the meta-analysis or other evidence synthesis? For Yes: included only low risk of bias RCTs OR, if the pooled estimate was based on RCTs and/or observational studies at variable RoB, the authors performed analyses to investigate possible impact of RoB on summary estimates of effect.

Item 13. Did the review authors account for RoB in individual studies when interpreting/ discussing the results of the review? For Yes: included only low risk of bias RCTs OR, if RCTs with moderate or high RoB, or NRSI were included the review provided a discussion of the likely impact of RoB on the results

Item 14. Did the review authors provide a satisfactory explanation for, and discussion of, any heterogeneity observed in the results of the review? For Yes: There was no significant heterogeneity in the results OR if heterogeneity was present the authors performed an investigation of sources of any heterogeneity in the results and discussed the impact of this on the results of the review

Item 15. If they performed quantitative synthesis did the review authors carry out an adequate investigation of publication bias (small study bias) and discuss its likely impact on the results of the review? For Yes: performed graphical or statistical tests for publication bias and discussed the likelihood and magnitude of impact of publication bias.

Item 16. Did the review authors report any potential sources of conflict of interest, including any funding they received for conducting the review? For Yes: The authors reported no competing interests OR The authors described their funding sources and how they managed potential conflicts of interest

Final Risk of Bias. Note: Multiple non-critical weaknesses may diminish confidence in the review and it may be appropriate to move the overall appraisal down from moderate to low confidence. Non-critical weakness = Q1, Q3, Q5, Q6, Q8, Q10, Q14, Q16 Critical weakness = Q2, Q4, Q7, Q11, Q15 Critical flaw = Q9, Q12, Q13

Details on the AMSTAR 2 critical appraisal tool are available from Shea et al.[1]

References:

1. Shea BJ, Reeves BC, Wells G, et al. AMSTAR 2: a critical appraisal tool for systematic reviews that include randomised or non-randomised studies of healthcare interventions, or both. BMJ. 2017;358. doi:10.1136/bmj.j4008

APPENDIX 4. Citation matrix of overlap of primary studies included in the systematic reviews and meta-analyses. (1) denotes a cited.

Study	Almousa 2019	Álvarez-García 2022	Alves 2025	Cerruto 2020	Culleton-Quinn 2022	de Mattos Lourenco 2017	Dominguez-Antuña 2023	Garcia-Perdomo 2022	Martins 2017	Pires 2020	Romero Parra 2024	Silva Pereira 2017	Syeda 2024	Teixeira 2018
Abitteboul et al. (2015)						**1**								
Almeida et al. (2016)	1				1	1								
Alves et al. (2017)	1													
Araujo et al. (2015)														1
Bø et al. (1994)	1								1					
Bø et al. (2001)				1		1								
Bø et al. (2004)						1								
Bø et al. (2010)				1		1								
Bø et al. (2011)				1		1			1					
Bogéa et al. (2018)		1												
Borin et al. (2013)										1		1		1
Cardoso et al. (2018)					1									
Carls et al. (2007)	1			1	1	1								
Carvalho et al. (2020)					1									
Caylet et al. (2006)				1	1	1								
Da Roza et al. (2013)				1										
Da Roza et al. (2015a)				1	1				1	1				
Da Roza et al. (2015b)	1			1										
Da Roza et al. (2015c)				1		1			1	1				
Da Roza, Brandão et al.(2015)	1													
Da Silva Souza et al.(2017)		1												
De Araujo (2020)		1												
De Araujo et al. (2008)				1		1	1							
De Machado et al. (2021)							1				1			
De Oliveira Leal et al.(2020)		1												

De Souza Pereira et al.(2019		1											
Di Benedetto et al. (2008)				1									
Dias et al. (2021)							1						
Dobrowolski et al. (2020)					1								
Dockter et al. (2007)	1				1								
Dos Santos Caldas et al.(2017)		1											
Dos Santos et al.(2009)									1				
Dos Santos et al.(2018)								1				1	
Eliasson et al. (2002)				1	1	1			1				
Eliasson et al. (2005)								1					
Eliasson et al. (2008)				1	1	1							
Eliasson Larsson, & Mattsson (2022)	1												
Elks et al.(2020)		1					1				1		
Elleuch et al. (1998)	1												
Faulks & Catto (2021)													
Ferreira et al.(2019)		1			1		1			1			
Forner et al. (2020)											1		
Fozzatti et al. (2012)	1			1		1		1					1
Gepharte et al. (2018)											1		
Gram & Bø (2020)					1								
Guimaraes (2019)		1											
Hagovska et al. (2017)	1				1			1		1			
Halvorsen et al. (1990)				1									
High et al.(2019)		1					1						
Huebner et al.(2022)			1										
Jácome et al. (2011)	1			1	1	1				1			1
Keefee et al. (1984)				1									
Kelecic et al. (2023)												1	

Khan et al.(2019)											1	
Khowailed et al. (2020)						1			1			
Kruger et al. (2007)	1											
Larsen & Yavorek (2006)				1								1
Leitner et al.(2017)								1				
Lopes et al. (2021)						1						
Ludviksdottir et al. (2018)	1			1								
Mahoney et al. (2023)											1	
Middlekauff et al. (2016)						1			1			
Nygaard et al. (1994)	1		1	1	1		1			1		
Nygaard et al. (1996)	1		1									
Nygaard et al. (1997)			1	1	1							
Parmigino et al. (2011)	1											
Patrizzi et al. (2014)							1					1
Pensin et al. (2012)							1					
Peters et al. (1999)			1									
Pires et al. (2020)				1							1	
Pisani et al. (2021)						1						
Pisani et al. (2022)						1						
Poświata et al. (2014)	1		1	1	1			1				1
Pozo-Municio et al.(2007)	1											
Regan (2016)		1										
Reis et al. (2011)	1									1		
Ribalta et al. (2010)								1				
Riddoch et al. (1988)			1									
Rodríguez-López et al. (2021)				1								
Rolli & Frigeri (2016)	1											
Salvatore et al. (2009)			1		1							

Sandwith & Robert (2021)					1					
Santos et al. (2009)	1									
Schettino et al. (2014)	1			1		1				
Silva dos Santos et al. (2009)				1						
Simeone et al. (2010)				1		1			1	1
Skaug et al. (2020)					1					
Skaug et al. (2022)			1		1					
Sullivan et al. (1992)				1						
Thyssen et al. (2002)				1	1	1			1	
Van der Worp et al. (2014)				1						
Velázquez-Saornil et al. (2021)					1					
Vitton et al. (2011)				1						
Wikander et al.(2019)		1	1				1			
Wikander et al.(2021a)			1							
Wikander et al.(2021b)					1					
Wikander et al.(2022)			1		1					
Yang et al.(2019)		1					1	1		
Yi et al. (2016)	1					1				

APPENDIX 5. Excluded primary studies after full-text review with reasons

1. Abitteboul Y, Leonard F, Mouly L, Riviere D, Oustric S. Incontinence urinaire chez des coureuses de loisir de marathon. *Progrès en Urologie*. 2015;25(11):636-641. doi:10.1016/j.purol.2015.05.009

Excluded: Age range (<18 years old, >45 years old).

2. Almeida MBA, Barra AA, Saltiel F, Silva-Filho AL, Fonseca AMRM, Figueiredo EM. Urinary incontinence and other pelvic floor dysfunctions in female athletes in Brazil: A cross-sectional study. *Scandinavian Med Sci Sports*. 2016;26(9):1109-1116. doi:10.1111/sms.12546

Excluded: Age range (<18 years old).

3. Altshuler PC, Burgard IM, Poling JK, Muffly T. Scaling the Heights: Rates of Pelvic Floor Dysfunction in Female Rock-Climbing Enthusiasts. *Wilderness & Environmental Medicine*. Published online November 25, 2024:10806032241290804. doi:10.1177/10806032241290804

Excluded: Age range (>45 years old).

4. Alves J, Luz S, Brandão S, Da Luz C, Jorge R, Da Roza T. Urinary Incontinence in Physically Active Young Women: Prevalence and Related Factors. *Int J Sports Med*. 2017;38(12):937-941. doi:10.1055/s-0043-115736

Excluded: Grouped sports, no individual disciplines.

5. Araújo MPD, Oliveira ED, Zucchi EVM, Trevisani VFM, Girão MJBC, Sartori MGF. Relação entre incontinência urinária em mulheres atletas corredoras de longa distância e distúrbio alimentar. *Rev Assoc Med Bras*. 2008;54(2):146-149. doi:10.1590/S0104-42302008000200018

Excluded: Age range (>45 years old).

6. Araujo MPD, Parmigiano TR, Negra LGD, et al. Avaliação do assoalho pélvico de atletas: existe relação com a incontinência urinária? *Rev Bras Med Esporte*. 2015;21(6):442-446. doi:10.1590/1517-869220152106140065

Excluded: Grouped sports, no individual disciplines.

7. Araujo MPD, Parmigiano TR, Negra LGD, et al. Evaluation of athletes' pelvic floor: is there a relation with urinary incontinence? *Rev Bras Med Esporte*. 2015;21(6):442-446. doi:10.1590/1517-869220152106140065

Excluded: Age range (<18 years old).

8. Arbieto ERM, Dos Santos KM, Da Luz SCT, Da Roza T. Comparison of urinary incontinence, based on pelvic floor and abdominal muscle strength, between nulliparous female athletes and non-athletes: A secondary analysis. *Neurourology and Urodynamics*. 2021;40(5):1140-1146. doi:10.1002/nau.24700

Excluded: Grouped sports, no individual disciplines.

9. Barbosa PN, Rodrigues MP, Costa E Silva ADA, Lemos CIL, Magno E Silva MP. Prevalence of urinary incontinence in Brazilian para athletes. *Br J Sports Med*. 2024;58(16):895-901. doi:10.1136/bjsports-2024-108076

Excluded: Para-athletes

10. Bennis S, Joyce C, Mueller ER, Fitzgerald CM. Individual Factors in Young Female Athletes' Bladder Health (the Y-FAB Study). *Urogynecology*. 2025;31(2):91-100. doi:10.1097/SPV.0000000000001576

Excluded: Grouped sports, no individual disciplines.

11. Bø K, Borgen JS. Prevalence of stress and urge urinary incontinence in elite athletes and controls: *Medicine and Science in Sports and Exercise*. 2001;33(11):1797-1802. doi:10.1097/00005768-200111000-00001

Excluded: Age range (<18 years old)

12. Bø K, Sundgot-Borgen J. Are former female elite athletes more likely to experience urinary incontinence later in life than non-athletes? *Scandinavian Med Sci Sports*. 2010;20(1):100-104. doi:10.1111/j.1600-0838.2008.00871.x

Excluded: Grouped sports, no individual disciplines.

13. Bonaldi L, Mascolini MV, Todesco M, et al. Urinary Incontinence and Other Pelvic Floor Dysfunctions as Underestimated Problems in People under Forty Years: What Is Their Relationship with Sport? *Life*. 2024;14(1). doi:10.3390/life14010067

Excluded: Grouped sports, no individual disciplines.

14. Bosch-Donate E, Vico-Moreno E, Fernández-Domínguez JC, González-Trujillo A, Sastre-Munar A, Romero-Franco N. Symptomatology and knowledge regarding pelvic floor dysfunctions and influence of gender stereotypes in female athletes. *Sci Rep*. 2024;14(1):11052. doi:10.1038/s41598-024-61464-x

Excluded: Age range (>45 years old).

15. Campbell KG, Batt ME, Drummond A. Prevalence of pelvic floor dysfunction in recreational athletes: a cross-sectional survey. *Int Urogynecol J*. 2023;34(10):2429-2437. doi:10.1007/s00192-023-05548-8

Excluded: Grouped sports, no individual disciplines.

16. Carls C. The prevalence of stress urinary incontinence in high school and college-age female athletes in the midwest: implications for education and prevention. *Urol Nurs*. 2007;27(1):21-39.

Excluded: Age range (<18 years old).

17. Carvalhais A, Natal Jorge R, Bø K. Performing high-level sport is strongly associated with urinary incontinence in elite athletes: a comparative study of 372 elite female athletes and 372 controls. *Br J Sports Med*. 2018;52(24):1586-1590. doi:10.1136/bjsports-2017-097587

Excluded: Age range (<18 years old).

18. Carvalho C, Da Silva Serrão PRM, Beleza ACS, Driusso P. Pelvic floor dysfunctions in female cheerleaders: a cross-sectional study. *Int Urogynecol J*. 2020;31(5):999-1006. doi:10.1007/s00192-019-04074-w

Excluded: Age range (<18 years old).

19. Da Roza T, Brandão S, Mascarenhas T, Jorge R, Duarte J. Urinary Incontinence and Levels of Regular Physical Exercise in Young Women. *Int J Sports Med*. 2015;36(09):776-780. doi:10.1055/s-0034-1398625

Excluded: Age range (<18 years old).

20. Da Roza T, Brandão S, Mascarenhas T, Jorge RN, Duarte JA. Volume of training and the ranking level are associated with the leakage of urine in young female trampolinists. *Clinical Journal of Sport Medicine*. 2015;25(3):270-275. doi:10.1097/JSM.0000000000000129

Excluded: Age range (<18 years old).

21. Da Roza T, Mascarenhas T, Araujo M, Trindade V, Jorge RN. Oxford Grading Scale vs manometer for assessment of pelvic floor strength in nulliparous sports students. *Physiotherapy*. 2013;99(3):207-211. doi:10.1016/j.physio.2012.05.014

Excluded: Grouped sports, no individual disciplines.

22. da Roza TD, Brandão S, Oliveira D, et al. Football practice and urinary incontinence: Relation between morphology, function and biomechanics. *Journal of Biomechanics*. 2015;48(9):1587-1592. doi:10.1016/j.jbiomech.2015.03.013

Excluded: Comparative study, incontinent vs. controls

23. Da Silva Pereira F, Haupenthal A, Da Roza TH, Mazo GZ, Virtuoso JF. Urine Loss during a Volleyball Competition: Comparison between Amateur and Professional Athletes. *PM&R*. 2021;13(10):1122-1126. doi:10.1002/pmrj.12502

Excluded: Age range (>45 years old).

24. Dakic JG, Cook J, Hay-Smith J, Lin KY, Frawley H. Pelvic floor disorders stop women exercising: A survey of 4556 symptomatic women. *Journal of Science and Medicine in Sport*. 2021;24(12):1211-1217. doi:10.1016/j.jsams.2021.06.003

Excluded: Age range (>45 years old).

25. Dakic JG, Hay-Smith J, Cook J, Lin KY, Frawley HC. Screening for pelvic floor symptoms in exercising women: a survey of 636 health and exercise professionals. *Journal of Science and Medicine in Sport*. 2023;26(2):80-86. doi:10.1016/j.jsams.2023.01.008

Excluded: Grouped sports, no individual disciplines.

26. Dobrowolski SL, Pudwell J, Harvey MA. Urinary incontinence among competitive rope-skipping athletes: a cross-sectional study. *Int Urogynecol J*. 2020;31(5):881-886. doi:10.1007/s00192-019-04048-y

Excluded: Age range (>45 years old).

27. Dos Santos KM, Da Roza T, Da Silva LL, Wolpe RE, Da Silva Honório GJ, Tonon Da Luz SC. Female sexual function and urinary incontinence in nulliparous athletes: An exploratory study. *Physical Therapy in Sport*. 2018;33:21-26. doi:10.1016/j.ptsp.2018.06.004

Excluded: Grouped sports, no individual disciplines.

28. Dos Santos KM, Da Roza T, Tonon Da Luz SC, Hort JP, Kruger JM, Schevchenco B. Quantification of Urinary Loss in Nulliparous Athletes During 1 Hour of Sports Training. *PM&R*. 2019;11(5):495-502. doi:10.1016/j.pmrj.2018.08.383

Excluded: Age range (>45 years old).

29. Eliasson K, Larsson T, Mattsson E. Prevalence of stress incontinence in nulliparous elite trampolinists. *Scandinavian Med Sci Sports*. 2002;12(2):106-110. doi:10.1034/j.1600-0838.2002.120207.x

Excluded: Age range (<18 years old)

30. Eliasson K, Nordlander I, Mattsson E, Larson B, Hammarström M. Prevalence of urinary leakage in nulliparous women with respect to physical activity and micturition habits. *International Urogynecology Journal and Pelvic Floor Dysfunction*. 2004;15(3):149-153. doi:10.1007/s00192-004-1135-y

Excluded: Grouped sports, no individual disciplines.

31. Elks W, Jaramillo-Huff A, Barnes KL, Petersen TR, Komesu YM. The Stress Urinary Incontinence in CrossFit (SUCCeSS) Study. *Female Pelvic Med Reconstr Surg*. 2020;26(2):101-106. doi:10.1097/SPV.0000000000000815

Excluded: Age range (>45 years old)

32. Elleuch M. L'incontinence urinaire chez la femme sportive nullipare. Enquete bpidbmiologique. A propos de 105 cas. *Annales de readaptation et de medecine physique*. Vol. 8. No. 41. 1998.

Excluded: Age range (<18 years old).

33. Figuers CC, Boyle KL, Caprio KM, Weidner AC. Pelvic Floor Muscle Activity and Urinary Incontinence in Weight-Bearing Female Athletes vs. Non-Athletes: *Journal of Women's Health Physical Therapy*. 2008;32(1):7-11. doi:10.1097/01274882-200832010-00002

Excluded: Grouped sports, no individual disciplines.

34. Forner LB, Beckman EM, Smith MD. Do women runners report more pelvic floor symptoms than women in CrossFit®? A cross-sectional survey. *Int Urogynecol J*. 2021;32(2):295-302. doi:10.1007/s00192-020-04531-x

Excluded: Age range (>45 years old).

35. Gan ZS, Bilger A, Smith AL. Urinary Incontinence in Nulliparous Female Elite Athletes: A Mixed Methods Exploration. *UROGC*. 2024;30(11):929-937. doi:10.1097/SPV.0000000000001573

Excluded: Grouped sports, no individual disciplines.

36. Gill N, Lin KY, Whitford E, Jeffrey S, Frawley H. The prevalence of urinary incontinence in nulliparous and parous adult netball players. *Continence*. 2025;13:101753. doi:10.1016/j.cont.2025.101753

Excluded: Age range (>45 years old).

37. Gram MCD, Bø K. High level rhythmic gymnasts and urinary incontinence: Prevalence, risk factors, and influence on performance. *Scandinavian Med Sci Sports*. 2020;30(1):159-165. doi:10.1111/sms.13548

Excluded: Age range (<18 years old).

38. Haakstad LAH, Gjestvang C, Lamerton T, Bø K. Urinary incontinence in a fitness club setting—is it a workout problem? *Int Urogynecol J*. 2020;31(9):1795-1802. doi:10.1007/s00192-020-04253-0

Excluded: Age range (>45 years old).

39. Hagovska M, Švihra J, Buková A, et al. Prevalence of Urinary Incontinence in Females Performing High-Impact Exercises. *Int J Sports Med*. 2017;38(03):210-216. doi:10.1055/s-0042-123045

Excluded: Grouped sports, no individual disciplines.

40. Hamilton HM, Mariano M, Kakar RS. Prevalence and Associated Factors of Urinary Incontinence in Female Recreational Runners. *Research Report*. Published online 2023.

Excluded: Age range (>45 years old).

41. High R, Thai K, Virani H, Kuehl T, Danford J. Prevalence of Pelvic Floor Disorders in Female CrossFit Athletes. *Female Pelvic Med Reconstr Surg*. 2020;26(8):498-502. doi:10.1097/SPV.0000000000000776

Excluded: Age range (>45 years old).

42. Huebner M, Ma W, Harding S. Sport-related risk factors for moderate or severe urinary incontinence in master female weightlifters: A cross-sectional study. Durkalec-Michalski K, ed. *PLoS ONE*. 2022;17(11):e0278376. doi:10.1371/journal.pone.0278376

Excluded: Age range (>45 years old).

43. Johnston CL, Negus MF, Rossiter MA, Phillips CG. A national survey of urinary incontinence in professional Team England female athletes. *European Journal of Obstetrics & Gynecology and Reproductive Biology*. 2023;282:12-16. doi:10.1016/j.ejogrb.2022.12.031

Excluded: Age range (>45 years old).

44. Kopyra K, Chomiuk T, Kasiak P, Mamcarz A, Śliż D. Evaluation of stress urinary incontinence in physically active and childless Polish females: a cross-sectional study. *Physiother Q*. 2024;32(3):80-85. doi:10.5114/pq/191514

Excluded: Grouped sports, no individual disciplines.

45. Kruger JA, Dietz HP, Murphy BA. Pelvic floor function in elite nulliparous athletes. *Ultrasound Obstet Gynecol*. 2007;30(1):81-85. doi:10.1002/uog.4027

Excluded: Grouped sports, no individual disciplines.

46. Larsen WI, Yavorek T. Pelvic prolapse and urinary incontinence in nulliparous college women in relation to paratrooper training. *Int Urogynecol J*. 2007;18(7):769-771. doi:10.1007/s00192-006-0226-3

Excluded: Grouped sports, no individual disciplines.

47. Larsen WI, Yavorek TA. Pelvic organ prolapse and urinary incontinence in nulliparous women at the United States Military Academy. *Int Urogynecol J*. 2006;17(3):208-210. doi:10.1007/s00192-005-1366-6

Excluded: Grouped sports, no individual disciplines.

48. Leitner M, Moser H, Eichelberger P, Kuhn A, Radlinger L. Evaluation of pelvic floor muscle activity during running in continent and incontinent women: An exploratory study. *Neurourol Urodyn*. 2017;36(6):1570-1576. doi:10.1002/nau.23151

Excluded: Age range (>45 years old).

49. Logan BL, Foster-Johnson L, Zotos E. Urinary incontinence among adolescent female athletes. *Journal of Pediatric Urology*. 2018;14(3):241.e1-241.e9. doi:10.1016/j.jpurol.2017.12.018

Excluded: Age range (<18 years old).

50. Mahoney K, Heidel RE, Olewinski L. Prevalence and Normalization of Stress Urinary Incontinence in Female Strength Athletes. *Journal of Strength and Conditioning Research*. 2023;37(9):1877-1881. doi:10.1519/JSC.0000000000004461

Excluded: Age range (>45 years old).

51. Moreno TRP, Araujo MPD, Benayon PC, Faroni RP, Barsottini CGN, Sartori MGF. Brazilian olympic female athletes' multidisciplinary care: an observational study. *Rev Bras Med Esporte*. 2022;28(4):281-285. doi:10.1590/1517-8692202228042021_0306

Excluded: Age range (<18 years old).

52. O'Halloran T, Bell RJ, Robinson PJ, Davis SR. Urinary incontinence in young nulligravid women: a cross-sectional analysis. *Ann Intern Med*. 2012;157(2):87-93. doi:10.7326/0003-4819-157-2-201207170-00005

Excluded: Age range (<18 years old).

53. Parmigiano TR, Zucchi EVM, Araujo MPD, et al. Pre-participation gynecological evaluation of female athletes: a new proposal. *Einstein (São Paulo)*. 2014;12(4):459-466. doi:10.1590/S1679-45082014AO3205

Excluded: Age range (<18 years old).

54. Pereira FDS, Haupenthal A, Arruda GTD, Virtuoso JF. Factors associated with urinary incontinence in volleyball athletes: a cross-sectional study. *Acta Sci Health Sci*. 2022;44:e58236. doi:10.4025/actascihealthsci.v44i1.58236

Excluded: Age range (<18 years old).

55. Pisani GK, De Oliveira Sato T, Carvalho C. Pelvic floor dysfunctions and associated factors in female CrossFit practitioners: a cross-sectional study. *Int Urogynecol J*. 2021;32(11):2975-2984. doi:10.1007/s00192-020-04581-1

Excluded: Data already included from nulliparous cohort.

56. Pozo-Municio C. Genitourinary conditions in young dancers: relationship between urinary incontinence and foot flexibility. *Journal of Dance Medicine & Science*. 2007;11(2):49-59. doi:10.1177/1089313X0701100204

Excluded: Age range (<18 years old).

57. Reis AO. Comparative study of the capacity of pelvic floor contraction in volleyball and basketball athletes. *Rev Bras Med Esporte*. 2011;17(2). 97-101. doi: 10.1590/S1517-86922011000200005

Excluded: Age range (<18 years old).

58. Rodríguez-López ES, Acevedo-Gómez MB, Romero-Franco N, et al. Urinary Incontinence Among Elite Track and Field Athletes According to Their Event Specialization: A Cross-Sectional Study. *Sports Med - Open*. 2022;8(1):78. doi:10.1186/s40798-022-00468-1

Excluded: Age range (<18 years old).

59. Rodríguez-López ES, Calvo-Moreno SO, Basas-García Á, Gutierrez-Ortega F, Guodemar-Pérez J, Acevedo-Gómez MB. Prevalence of urinary incontinence among elite athletes of both sexes. *Journal of Science and Medicine in Sport*. 2021;24(4):338-344. doi:10.1016/j.jsams.2020.09.017

Excluded: Age range (<18 years old).

60. Rohde M, Brumitt J, J S, Bø K. Prevalence of stress urinary incontinence in elite female endurance athletes. *J Hum Kinet*. 2025;23(3):Article 31.

Excluded: Age range (>45 years old).

61. Rolli F, Frigeri D. A prospective comparative urinary incontinence study between nulliparous female basketball athletes and non-athletes indicates a key role for health professionals in prevention.

Excluded: Age range (<18 years old).

62. Rubin N, Cerentini TM, Schlöttgen J, et al. Urinary incontinence and quality of life in high-performance swimmers: An observational study. *Health Care Women Int*. 2024;45(12):1446-1455. doi:10.1080/07399332.2023.2197861

Excluded: Comparative study, incontinent vs. controls

63. Sade S, Naor I, Rotem R, Waichenberg L, Kravits DZ, Weintraub AY. Pelvic floor disorders among amateur runners. *Arch Gynecol Obstet*. 2024;309(5):2223-2228. doi:10.1007/s00404-023-07351-8

Excluded: Age range (>45 years old).

64. Salvatore S, Serati M, Laterza R, Uccella S, Torella M, Bolis PF. The impact of urinary stress incontinence in young and middle-age women practising recreational sports activity: an epidemiological study. *Br J Sports Med*. 2009;43(14):1115-1118. doi:10.1136/bjsm.2008.049072

Excluded: Grouped sports, no individual disciplines.

65. Salvo CJ, Crewe A, Estes D, Kroboth J, Yost C. Screening for Incidence and Effect of Pelvic Floor Dysfunction in College-Aged Athletes. *International Journal of Sports Physical Therapy*. 2024;19(7). doi:10.26603/001c.120211

Excluded: Grouped sports, no individual disciplines.

66. Schwamberger T, da Roza TH, Arbieto ERM, et al. Triathletes and Urinary Incontinence: An Investigation of Prevalence and Associated Factors. *Neurourol Urodyn*. Published online 2025. doi:10.1002/nau.70145

Excluded: Age range (>45 years old).

67. Schettino MT, Mainini G, Ercolano S, et al. Risk of pelvic floor dysfunctions in young athletes. *Clin Exp Obstet Gynecol*. 2014;41(6):671-676. doi:10.12891/ceog19382014

Excluded: Age range (<18 years old)

68. Selecka M, Hagovska M, Bukova A, Svihra J. Influence of sports groups on the risk of stress urinary incontinence in sportswomen. *Eur J Obstet Gynecol Reprod Biol*. 2021;264:374-379. doi:10.1016/j.ejogrb.2021.07.047

Excluded: Age range (<18 years old)

69. Simeone C, Moroni A, Pettenò A, et al. Occurrence Rates and Predictors of Lower Urinary Tract Symptoms and Incontinence in Female Athletes. *Urologia*. 2010;77(2):139-146. doi:10.1177/039156031007700210

Excluded: Age range (>45 years old).

70. Skaug KL, Engh ME, Frawley H, Bø K. Prevalence of Pelvic Floor Dysfunction, Bother, and Risk Factors and Knowledge of the Pelvic Floor Muscles in Norwegian Male and Female Powerlifters and Olympic Weightlifters. *Journal of Strength and Conditioning Research*. 2022;36(10):2800-2807. doi:10.1519/JSC.0000000000003919

Excluded: Age range (>45 years old).

71. Skaug KL, Engh ME, Frawley H, Bø K. Urinary and anal incontinence among female gymnasts and cheerleaders—bother and associated factors. A cross-sectional study. *Int Urogynecol J*. 2022;33(4):955-964. doi:10.1007/s00192-021-04696-z

Excluded: Age range (>45 years old).

72. Stickley L, McDowell D. Prevalence, Knowledge, and Perceived Impact of Urinary Incontinence in Female Collegiate Athletes. *Journal of Women's & Pelvic Health Physical Therapy*. 2023;47(4):237-244. doi:10.1097/JWH.0000000000000274

Excluded: Grouped sports, no individual disciplines.

73. Syeda F, Pandit U. Prevalence of urinary incontinence in nulliparous Indian sportswomen. *J Fam Med Prim Care*. 2024;13(8):3270-3274. doi: 10.4103/jfmpc.jfmpc_282_24

Excluded: Age range (<18 years old).

74. Thyssen HH, Clevin L, Olesen S, Lose G. Urinary Incontinence in Elite Female Athletes and Dancers. *Int Urogynecol J*. 2002;13(1):15-17. doi:10.1007/s001920200003

Excluded: Age range (>45 years old).

75. Velázquez-Saornil J, Méndez-Sánchez E, Gómez-Sánchez S, et al. Observational Study on the Prevalence of Urinary Incontinence in Female Athletes. *IJERPH*. 2021;18(11):5591. doi:10.3390/ijerph18115591

Excluded: Age range not specified.

76. Vitton V, Baumstarck-Barrau K, Brardjanian S, Caballe I, Bouvier M, Grimaud JC. Impact of High-Level Sport Practice on Anal Incontinence in a Healthy Young Female Population. *Journal of Women's Health*. 2011;20(5):757-763. doi:10.1089/jwh.2010.2454

Excluded: Grouped sports, no individual disciplines.

77. Warzecha M, Milka W, Paluch K, Pępek A, Amarowicz J. The frequency and severity of urinary incontinence symptoms in the group of adult physically active women. *Fiz Pol*. 2024;24(5):6-16. doi:10.56984/8ZG020CH32F

Excluded: Age range (>45 years old).

78. Whitney KE, Holtzman B, Cook D, et al. Low energy availability and impact sport participation as risk factors for urinary incontinence in female athletes. *Journal of Pediatric Urology*. 2021;17(3):290.e1-290.e7. doi:10.1016/j.jpurol.2021.01.041

Excluded: Age range (<18 years old).

79. Wikander L, Cross D, Gahreman DE. Prevalence of urinary incontinence in women powerlifters: a pilot study. *Int Urogynecol J*. 2019;30(12):2031-2039. doi:10.1007/s00192-019-03870-8

Excluded: Age range (>45 years old).

80. Wikander L, Kirshbaum MN, Waheed N, Gahreman DE. Urinary Incontinence in Competitive Women Powerlifters: A Cross-Sectional Survey. *Sports Med - Open*. 2021;7(1):89. doi:10.1186/s40798-021-00387-7

Excluded: Age range (>45 years old).

81. Wikander L, Kirshbaum MN, Waheed N, Gahreman DE. Urinary Incontinence in Competitive Women Weightlifters. *Journal of Strength and Conditioning Research*. 2022;36(11):3130-3135. doi:10.1519/JSC.0000000000004052

Excluded: Age range (>45 years old).

82. Yang J, Cheng JW, Wagner H, et al. The effect of high impact crossfit exercises on stress urinary incontinence in physically active women. *Neurourology and Urodynamics*. 2019;38(2):749-756. doi:10.1002/nau.23912

Excluded: Age range (>45 years old).

83. Yi J, Tenfelde S, Tell D, Brincat C, Fitzgerald C. Triathlete Risk of Pelvic Floor Disorders, Pelvic Girdle Pain, and Female Athlete Triad. *Female Pelvic Med Reconstr Surg*. 2016;22(5):373-376. doi:10.1097/SPV.0000000000000296

Excluded: Age range (>45 years old)

APPENDIX 6. Appraisal tool for Cross-Sectional Studies (AXIS) of included primary studies.

Study	# 1	# 2	# 3	# 4	# 5	# 6	# 7	# 8	# 9	# 10	# 11	# 12	# 13	# 14	# 15	# 16	# 17	# 18	# 19	# 20	Score	Global rating
Araujo 2019	Y	Y	N	Y	P	N	N	Y	Y	Y	Y	Y	N	N	Y	Y	Y	N	Y	Y	14.5	Moderate
Cardoso, 2018	Y	Y	Y	Y	Y	P	P	Y	Y	Y	Y	Y	Y	P	Y	Y	Y	P	Y	Y	18.0	High
Caylet, 2006	Y	Y	Y	Y	P	P	N	Y	Y	Y	Y	Y	P	N	Y	Y	Y	Y	Y	Y	16.0	High
Clapin, 2025	Y	Y	N	Y	Y	N	P	Y	Y	Y	Y	Y	N	Y	Y	Y	Y	Y	Y	P	16.0	High
Culleron-Quin, 2024	Y	Y	N	Y	Y	N	P	Y	Y	Y	Y	Y	N	Y	Y	Y	Y	Y	Y	Y	16.5	High
Cygańska 2025	Y	Y	Y	Y	Y	P	P	Y	Y	Y	Y	Y	P	N	Y	Y	Y	Y	Y	Y	18.0	High
da Silva Borin, 2013	Y	Y	Y	Y	P	N	N	Y	P	Y	Y	Y	N	N	Y	Y	Y	Y	Y	Y	15.0	Moderate
Dakic 2025	Y	Y	Y	Y	Y	P	P	Y	Y	Y	Y	Y	Y	P	Y	Y	Y	Y	Y	Y	18.0	High
da Silva Pereira, 2021	Y	Y	N	Y	P	N	N	Y	Y	Y	Y	Y	N	N	Y	Y	Y	Y	Y	Y	15.0	Moderate
de Souza Pereira 2022	Y	Y	Y	Y	P	P	N	Y	Y	Y	Y	Y	N	N	Y	Y	Y	Y	Y	Y	16.0	High
Dockter, 2007	Y	Y	N	Y	P	N	N	Y	N	Y	Y	Y	N	N	Y	Y	Y	Y	N	Y	12.5	Moderate
dos Santos, 2009	Y	Y	N	Y	P	P	P	Y	N	Y	N	Y	P	Y	Y	Y	Y	N	Y	Y	14.0	Moderate
Faulks 2021	Y	Y	N	Y	P	N	N	Y	N	N	Y	Y	N	N	Y	Y	Y	Y	Y	Y	12.5	Moderate
Fozzatti, 2012	Y	Y	Y	Y	P	N	N	Y	Y	Y	Y	Y	N	N	Y	Y	Y	P	Y	Y	15.0	Moderate
Gan, 2025	Y	Y	N	Y	P	N	N	Y	P	Y	Y	Y	N	N	Y	Y	Y	Y	Y	Y	14.0	Moderate
Gill, 2025	Y	Y	Y	Y	Y	P	P	Y	P	Y	Y	Y	Y	Y	Y	Y	Y	Y	Y	Y	18.5	High
Hagovska, 2018	Y	Y	Y	Y	P	P	N	Y	Y	Y	Y	Y	P	Y	Y	N	Y	Y	Y	Y	16.5	High
Jacome 2011	Y	Y	N	Y	P	N	N	Y	N	Y	Y	Y	N	N	Y	Y	Y	Y	Y	Y	13.5	Moderate
Kelečić, 2023	Y	Y	N	Y	N	N	N	Y	Y	Y	Y	Y	N	N	Y	Y	Y	Y	Y	Y	14.0	Moderate
Lopes, 2020	Y	Y	N	Y	P	N	P	Y	Y	Y	Y	Y	Y	Y	Y	Y	Y	Y	Y	Y	17.0	High
Machado, 2021	Y	Y	Y	Y	P	N	N	Y	Y	Y	Y	Y	N	N	Y	Y	Y	Y	Y	Y	15.5	Moderate
McCarthy-Ryan, 2024	Y	Y	N	Y	N	P	N	Y	Y	Y	Y	Y	N	Y	Y	Y	Y	Y	Y	Y	15.5	Moderate
Middlekauff, 2016	Y	Y	Y	Y	P	N	N	Y	Y	Y	Y	Y	N	N	Y	Y	Y	Y	Y	Y	15.5	Moderate
Parr, 2023	Y	Y	N	Y	P	N	P	Y	Y	Y	Y	Y	P	Y	Y	Y	Y	Y	P	Y	16.0	High
Patrizzi, 2014	Y	Y	N	Y	P	N	N	Y	N	Y	Y	Y	N	N	Y	Y	Y	Y	N	Y	12.5	Moderate
Pisani, 2022	Y	Y	N	Y	N	N	N	Y	Y	Y	Y	Y	N	N	Y	Y	Y	Y	Y	Y	14.0	Moderate
Poświata, 2014	Y	Y	N	Y	Y	P	N	Y	Y	Y	Y	Y	P	N	Y	Y	Y	Y	N	Y	14.0	Moderate
Sandwith, 2021	Y	Y	N	Y	Y	Y	Y	Y	Y	Y	Y	Y	Y	Y	Y	Y	Y	Y	Y	Y	19.0	High
Sebastian Rico, 2024	Y	Y	Y	Y	Y	N	N	Y	Y	Y	Y	Y	N	N	Y	Y	Y	Y	Y	Y	16.0	High
Tarczewska, 2024	Y	Y	N	Y	P	N	N	Y	P	N	Y	Y	N	N	Y	Y	Y	Y	N	Y	12.0	Moderate
Valastro, 2025	Y	Y	Y	Y	Y	P	N	Y	Y	Y	Y	Y	N	N	Y	Y	Y	Y	Y	Y	16.5	High
Winder 2023	Y	Y	N	Y	P	N	N	Y	Y	Y	Y	Y	N	N	Y	Y	Y	Y	Y	Y	15.0	Moderate

Notes:
Item 1. Were the aims/objectives of the study clear?
Item 2. Was the study design appropriate for the stated aim(s)?

Item 3. Was the sample size justified?
Item 4. Was the target/reference population clearly defined? (Is it clear who the research was about?)
Item 5. Was the sample frame taken from an appropriate population base so that it closely represented the target/reference population under investigation?
Item 6. Was the selection process likely to select subjects/participants that were representative of the target/reference population under investigation?
Item 7. Were measures undertaken to address and categorise non-responders?
Item 8. Were the risk factor and outcome variables measured appropriate to the aims of the study?
Item 9. Were the risk factor and outcome variables measured correctly using instruments/measurements that had been trialled, piloted or published previously?
Item 10. Is it clear what was used to determine statistical significance and/or precision estimates (e.g., p-values, confidence intervals)?
Item 11. Were the methods (including statistical methods) sufficiently described to enable them to be repeated?
Item 12. Were the basic data adequately described?
Item 13. Does the response rate raise concerns about non-response bias? (reverse-coded)
Item 14. If appropriate, was information about non-responders described?
Item 15. Were the results internally consistent?
Item 16. Were the results presented for all the analyses described in the methods?
Item 17. Were the authors' discussions and conclusions justified by the results?
Item 18. Were the limitations of the study discussed?
Item 19. Were there any funding sources or conflicts of interest that may affect the authors' interpretation of the results?
Item 20. Was ethical approval or consent of participants attained

Items were coded Yes(Y)=1, Partial(P)=0.5, No(N)=0, per AXIS guidance; total scores were mapped to Global rating as follows: High ≥16.5; Moderate 13–16; Low <13 (thresholds pre-specified). Details on the AXIS tool are available from Downes et al.[2]

References:

1. Downes MJ, Brennan ML, Williams HC, Dean RS. Development of a critical appraisal tool to assess the quality of cross-sectional studies (AXIS). *BMJ Open*. 2016;6(12):e011458. doi:10.1136/bmjopen-2016-011458

APPENDIX 7. Publication bias assessed by Egger's regression test and funnel plots for subgroup meta-analyses.

Competitive level: t = 1.29, df = 31, p-value = 0.2065; Bias estimate: 1.9378 (SE = 1.5017)

Sport impact = -0.54, df = 49, p-value = 0.5923; Bias estimate: -0.5156 (SE = 0.9564)

Sport modality: t = -1.25, df = 57, p-value = 0.2164; Bias estimate: -0.8588 (SE = 0.6871)

Sport discipline: t = -2.06, df = 71, p-value = 0.0427; Bias estimate: -0.7662 (SE = 0.3713)

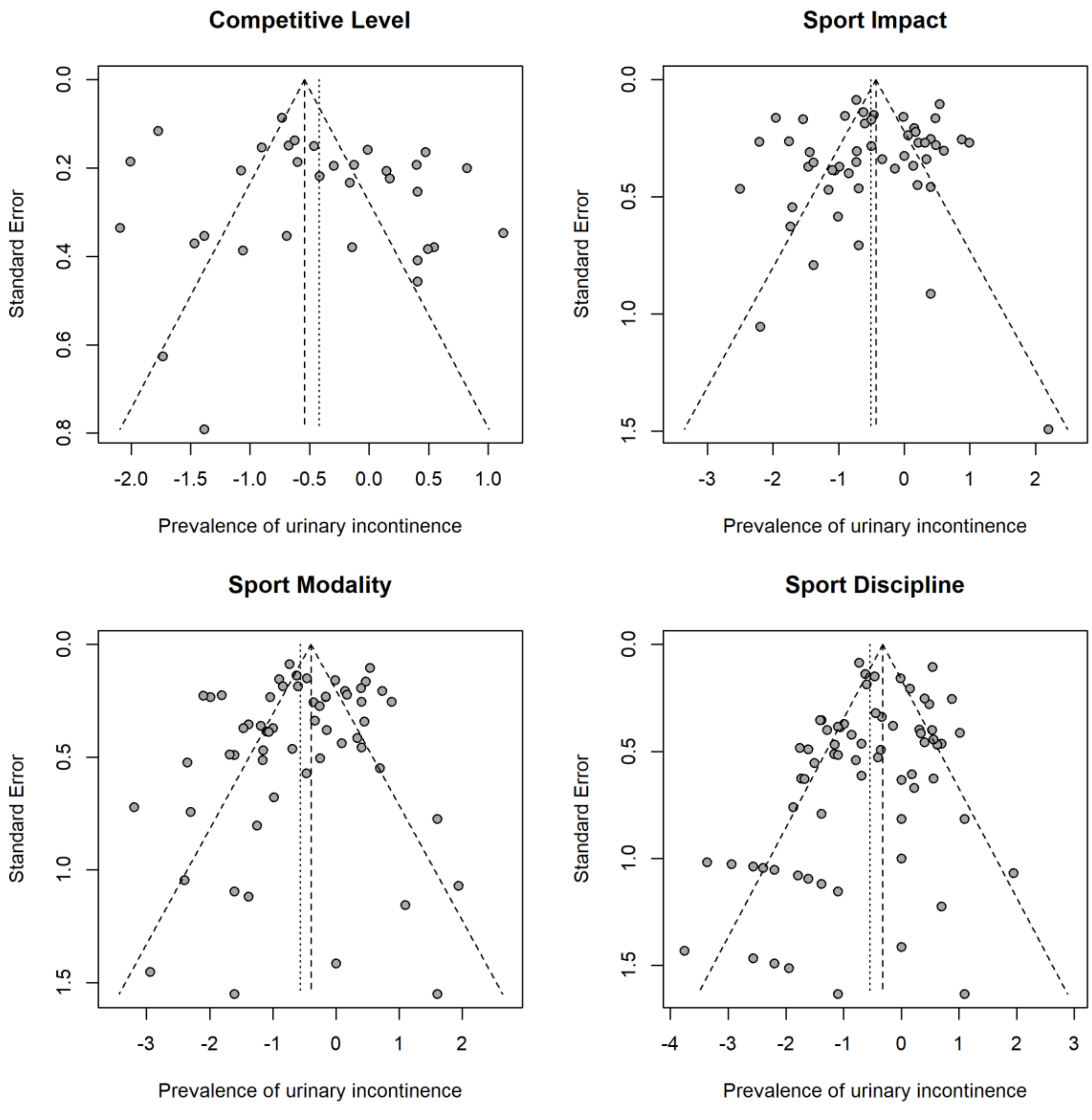

APPENDIX 8. Sport characteristics, urinary incontinence outcomes, and training exposure of each cohort included in the meta-analysis

Study	Level of competition	Sport impact	Sport discipline	Sport modality	UI			SUI			UUI			MUI			EXUI			Training volume (h/wk)
					Cases	Total	%	Cases	Total	%	Cases	Total	%	Cases	Total	%	Cases	Total	%	
Araujo, 2019	Amateur	High-impact	CrossFit	Weight	45	127	35%	–	–	–	–	–	–	–	–	–	–	–	–	–
Cardoso, 2018	High-level	High-impact	Basketball	Ball Games	14	21	67%	–	–	–	–	–	–	–	–	–	–	–	–	–
Cardoso, 2018	High-level	High-impact	Volleyball	Ball Games	17	27	63%	–	–	–	–	–	–	–	–	–	–	–	–	–
Cardoso, 2018	High-level	Medium-impact	Futsal	Ball Games	20	30	67%	–	–	–	–	–	–	–	–	–	–	–	–	–
Cardoso, 2018	High-level	Medium-impact	Handball	Ball Games	22	30	73%	–	–	–	–	–	–	–	–	–	–	–	–	–
Cardoso, 2018	High-level	Medium-impact	Athletics	Endurance	7	8	88%	–	–	–	–	–	–	–	–	–	–	–	–	–
Cardoso, 2018	High-level	Medium-impact	Judo	Weight	2	2	100%	–	–	–	–	–	–	–	–	–	–	–	–	–
Caylet, 2006	Professional	Medium-impact	Tennis	Ball Games	1	12	8%	–	–	–	–	–	–	–	–	–	–	–	–	9.3
Caylet, 2006	Professional	Medium-impact	Soccer	Ball Games	2	15	13%	–	–	–	–	–	–	–	–	–	–	–	–	9.3
Caylet, 2006	Professional	Medium-impact	Handball	Ball Games	5	16	31%	–	–	–	–	–	–	–	–	–	–	–	–	9.3
Caylet, 2006	Professional	Medium-impact	Rugby	Ball Games	5	16	31%	–	–	–	–	–	–	–	–	–	–	–	–	9.3
Caylet, 2006	Professional	High-impact	Volleyball	Ball Games	7	17	41%	–	–	–	–	–	–	–	–	–	–	–	–	9.3
Caylet, 2006	Professional	High-impact	Basketball	Ball Games	5	20	25%	–	–	–	–	–	–	–	–	–	–	–	–	9.3
Caylet, 2006	Professional	Low-impact	Swimming	Endurance	7	21	33%	–	–	–	–	–	–	–	–	–	–	–	–	9.3
Caylet, 2006	Professional	Medium-impact	Combat sports	Weight	0	9	0%	–	–	–	–	–	–	–	–	–	–	–	–	9.3
Clapin, 2025	Professional	Medium-impact	Rugby	Ball Games	79	159	50%	48	159	30%	93	147	63%	49	159	31%	–	–	–	13.9
Culleton Quinn, 2024	Professional	Medium-impact	Camogie	Ball Games	47	79	59%	26	79	33%	5	79	6%	14	79	18%	–	–	–	7.8
Culleton Quinn, 2024	Professional	Medium-impact	Gaelic football	Ball Games	51	80	64%	25	80	31%	2	80	3%	17	80	21%	–	–	–	8.5
Cygańska, 2025	High-level	High-impact	Horse jumpers	Technical	9	48	19%	9	48	19%	7	48	15%	–	–	–	–	–	–	3.3
da Silva Borin, 2013	Amateur	Medium-impact	Handball	Ball Games	2	10	20%	–	–	–	–	–	–	–	–	–	1	10	10%	8
da Silva Borin, 2013	High-level	High-impact	Volleyball	Ball Games	2	10	20%	–	–	–	–	–	–	–	–	–	2	10	20%	17
da Silva Borin, 2013	High-level	High-impact	Basketball	Ball Games	1	10	10%	–	–	–	–	–	–	–	–	–	2	10	20%	25
da Silva Pereira, 2021	Amateur	High-impact	Volleyball	Ball Games	19	30	63%	–	–	–	–	–	–	–	–	–	15	30	50%	6
da Silva Pereira, 2021	Professional	High-impact	Volleyball	Ball Games	34	45	76%	–	–	–	–	–	–	–	–	–	25	45	56%	12
Dakic, 2025	Professional	Medium-impact	Rugby	Ball Games	13	28	46%	8	28	29%	2	28	7%	3	28	11%	–	–	–	–
de Souza Pereira, 2022	Amateur	High-impact	CrossFit	Weight	73	189	39%	51	189	27%	6	189	3%	12	189	6%	–	–	–	4.4
Dockter, 2007	High-level	High-impact	Cheerleaders	Aesthetic	2	9	22%	4	22	18%	2	22	9%	–	–	–	4	22	18%	–
Dockter, 2007	High-level	Medium-impact	Softball	Ball Games	4	12	33%	4	12	33%	3	12	25%	–	–	–	5	12	42%	–
Dockter, 2007	High-level	Medium-impact	Soccer	Ball Games	13	20	65%	2	9	22%	1	9	11%	–	–	–	2	9	22%	–
Dockter, 2007	High-level	High-impact	Basketball	Ball Games	4	22	18%	14	22	64%	9	22	41%	–	–	–	9	22	41%	–
Dockter, 2007	High-level	High-impact	Volleyball	Ball Games	14	22	64%	13	20	65%	4	20	20%	–	–	–	10	20	50%	–
Dockter, 2007	High-level	Medium-impact	Track and field	Endurance	14	24	58%	14	24	58%	13	24	54%	–	–	–	14	24	58%	–
dos Santos, 2009	Amateur	Low-impact	Water aerobics	Aesthetic	0	3	0%	–	–	–	–	–	–	–	–	–	–	–	–	–
dos Santos, 2009	Amateur	Low-impact	Water aerobics	Aesthetic	1	4	25%	–	–	–	–	–	–	–	–	–	–	–	–	–
dos Santos, 2009	Amateur	High-impact	Trampoline	Aesthetic	2	9	22%	–	–	–	–	–	–	–	–	–	–	–	–	–
dos Santos, 2009	Amateur	High-impact	Gymnastics	Aesthetic	1	30	3%	–	–	–	–	–	–	–	–	–	–	–	–	–
dos Santos, 2009	Amateur	Medium-impact	Tennis	Ball Games	0	1	0%	–	–	–	–	–	–	–	–	–	–	–	–	–
dos Santos, 2009	Amateur	Medium-impact	Soccer	Ball Games	0	4	0%	–	–	–	–	–	–	–	–	–	–	–	–	–
dos Santos, 2009	Amateur	Medium-impact	Handball	Ball Games	1	5	20%	–	–	–	–	–	–	–	–	–	–	–	–	–

dos Santos, 2009	Amateur	High-impact	Basketball	Ball Games	1	6	17%	–	–	–	–	–	–	–	–	–	–	–	–	–
dos Santos, 2009	Amateur	High-impact	Volleyball	Ball Games	0	6	0%	–	–	–	–	–	–	–	–	–	–	–	–	–
dos Santos, 2009	Amateur	Low-impact	Cycling	Endurance	0	2	0%	–	–	–	–	–	–	–	–	–	–	–	–	–
dos Santos, 2009	Amateur	High-impact	Rope jumping	Endurance	0	3	0%	–	–	–	–	–	–	–	–	–	–	–	–	–
dos Santos, 2009	Amateur	Low-impact	Swimming	Endurance	3	6	50%	–	–	–	–	–	–	–	–	–	–	–	–	–
dos Santos, 2009	Amateur	High-impact	Weightlifting	Weight	1	12	8%	–	–	–	–	–	–	–	–	–	–	–	–	–
Faulks, 2021	Professional	Medium-impact	Rugby	Ball Games	39	65	60%	–	–	–	–	–	–	–	–	–	39	65	60%	–
Fozzatti, 2012	Amateur	Low-impact	Water aerobics	Aesthetic	10	43	23%	–	–	–	–	–	–	–	–	–	23	164	14%	3.7
Fozzatti, 2012	Amateur	Low-impact	Swimming	Endurance	1	14	7%	–	–	–	–	–	–	–	–	–	10	43	23%	3.7
Fozzatti, 2012	Amateur	Low-impact	Cycling	Endurance	9	89	10%	–	–	–	–	–	–	–	–	–	1	7	14%	3.7
Fozzatti, 2012	Amateur	Low-impact	Hiking	Endurance	12	99	12%	–	–	–	–	–	–	–	–	–	9	68	13%	3.7
Fozzatti, 2012	Amateur	High-impact	Bodybuilding	Weight	23	164	14%	–	–	–	–	–	–	–	–	–	2	36	6%	3.7
Fozzatti, 2012	Amateur	Low-impact	Hydrogymnastic	NC	1	7	14%	–	–	–	–	–	–	–	–	–	20	80	25%	3.7
Fozzatti, 2012	Amateur	Low-impact	Pilates	NC	2	36	6%	–	–	–	–	–	–	–	–	–	17	87	20%	3.7
Fozzatti, 2012	Amateur	Low-impact	Muscle stretching	NC	9	68	13%	–	–	–	–	–	–	–	–	–	14	73	19%	3.7
Fozzatti, 2012	Amateur	High-impact	Jump	Gravity	20	80	25%	–	–	–	–	–	–	–	–	–	1	14	7%	3.7
Gan, 2025	Professional	Medium-impact	Diving	Aesthetic	1	1	100%	–	–	–	–	–	–	–	–	–	–	–	–	–
Gan, 2025	Professional	High-impact	Gymnastics	Aesthetic	2	3	67%	–	–	–	–	–	–	–	–	–	–	–	–	–
Gan, 2025	Professional	High-impact	Basketball	Ball Games	1	1	100%	–	–	–	–	–	–	–	–	–	–	–	–	–
Gan, 2025	Professional	Medium-impact	Field hockey	Ball Games	1	1	100%	–	–	–	–	–	–	–	–	–	–	–	–	–
Gan, 2025	Professional	High-impact	Volleyball	Ball Games	0	1	0%	–	–	–	–	–	–	–	–	–	–	–	–	–
Gan, 2025	Professional	Medium-impact	Lacrosse	Ball Games	1	2	50%	–	–	–	–	–	–	–	–	–	–	–	–	–
Gan, 2025	Professional	Medium-impact	Softball	Ball Games	1	2	50%	–	–	–	–	–	–	–	–	–	–	–	–	–
Gan, 2025	Professional	Medium-impact	Squash	Ball Games	0	2	0%	–	–	–	–	–	–	–	–	–	–	–	–	–
Gan, 2025	Professional	Medium-impact	Soccer	Ball Games	1	4	25%	–	–	–	–	–	–	–	–	–	–	–	–	–
Gan, 2025	Professional	Low-impact	Rowing	Endurance	4	4	100%	–	–	–	–	–	–	–	–	–	–	–	–	–
Gan, 2025	Professional	Medium-impact	Track and field	Endurance	6	8	75%	–	–	–	–	–	–	–	–	–	–	–	–	–
Gill, 2025	High-level	Medium-impact	Netball	Ball Games	44	81	54%	27	81	33%	10	81	12%	–	–	–	–	–	–	–
Hagovska, 2018	Amateur	High-impact	Dance	Aesthetic	2	31	6%	2	31	6%	–	–	–	–	–	–	–	–	–	5.9
Hagovska, 2018	Amateur	Medium-impact	Figure skating	Aesthetic	0	20	0%	0	20	0%	–	–	–	–	–	–	–	–	–	8.3
Hagovska, 2018	Amateur	Medium-impact	Tennis	Ball Games	0	21	0%	0	21	0%	–	–	–	–	–	–	–	–	–	6.5
Hagovska, 2018	Amateur	Medium-impact	Floorball	Ball Games	0	18	0%	0	18	0%	–	–	–	–	–	–	–	–	–	6.5
Hagovska, 2018	Amateur	Medium-impact	Soccer	Ball Games	1	20	5%	1	20	5%	–	–	–	–	–	–	–	–	–	7.7
Hagovska, 2018	Amateur	High-impact	Volleyball	Ball Games	10	51	20%	10	51	20%	–	–	–	–	–	–	–	–	–	8.8
Hagovska, 2018	Amateur	Medium-impact	Handball	Ball Games	5	30	17%	5	30	17%	–	–	–	–	–	–	–	–	–	10.4
Hagovska, 2018	Amateur	High-impact	Basketball	Ball Games	5	34	15%	5	34	15%	–	–	–	–	–	–	–	–	–	10.9
Hagovska, 2018	Amateur	Medium-impact	Athletics	Endurance	5	21	24%	5	21	24%	–	–	–	–	–	–	–	–	–	9
Hagovska, 2018	Amateur	Medium-impact	Fitness	Weight	5	32	16%	5	32	16%	–	–	–	–	–	–	–	–	–	9.3
Jácome, 2011	Amateur	High-impact	Basketball	Ball Games	15	36	42%	–	–	–	–	–	–	–	–	–	–	–	–	–
Jácome, 2011	Amateur	Medium-impact	Futsal	Ball Games	19	38	50%	–	–	–	–	–	–	–	–	–	–	–	–	–
Kelečić, 2023	Professional	High-impact	Gymnastics	Aesthetic	0	3	0%	–	–	–	–	–	–	–	–	–	–	–	–	16
Kelečić, 2023	Professional	Low-impact	Synchronised swimming	Aesthetic	7	13	54%	–	–	–	–	–	–	–	–	–	–	–	–	16
Kelečić, 2023	Professional	High-impact	Volleyball	Ball Games	2	4	50%	–	–	–	–	–	–	–	–	–	–	–	–	16
Kelečić, 2023	Professional	Low-impact	Table tennis	Ball Games	3	6	50%	–	–	–	–	–	–	–	–	–	–	–	–	16

Kelečić, 2023	Professional	Medium-impact	Soccer	Ball Games	1	7	14%	–	–	–	–	–	–	–	–	–	–	–	–	16
Kelečić, 2023	Professional	Low-impact	Waterpolo	Ball Games	5	8	63%	–	–	–	–	–	–	–	–	–	–	–	–	16
Kelečić, 2023	Professional	Medium-impact	Tennis	Ball Games	7	11	64%	–	–	–	–	–	–	–	–	–	–	–	–	16
Kelečić, 2023	Professional	Medium-impact	Handball	Ball Games	8	27	30%	–	–	–	–	–	–	–	–	–	–	–	–	16
Kelečić, 2023	Professional	Low-impact	Swimming	Endurance	1	1	100%	–	–	–	–	–	–	–	–	–	–	–	–	16
Kelečić, 2023	Professional	Medium-impact	Athletics	Endurance	0	4	0%	–	–	–	–	–	–	–	–	–	–	–	–	16
Kelečić, 2023	Professional	High-impact	Weightlifting	Weight	1	2	50%	–	–	–	–	–	–	–	–	–	–	–	–	16
Kelečić, 2023	Professional	Low-impact	Alpine skiing	Technical	0	2	0%	–	–	–	–	–	–	–	–	–	–	–		16
Lopes, 2020	Amateur	High-impact	CrossFit	Weight	10	50	20%	1	50	2%	1	50	2%	–	–	–	–	–	–	5.2
Machado, 2021	Amateur	High-impact	CrossFit	Weight	12	20	60%	–	–	–	–	–	–	–	–	–	–	–	–	4.5
McCarthy-Ryan, 2024	NR	Medium-impact	Rugby	Ball Games	250	396	63%	250	396	63%	–	–	–	–	–	–	172	396	43%	–
Middlekauff, 2016	Amateur	High-impact	CrossFit	Weight	9	35	26%	–	–	–	–	–	–	–	–	–	–	–	–	4.3
Parr, 2023	High-level	High-impact	Cheerleaders	Aesthetic	1	2	50%	–	–	–	–	–	–	–	–	–	–	–	–	14.9
Parr, 2023	High-level	High-impact	Basketball	Ball Games	5	9	56%	–	–	–	–	–	–	–	–	–	–	–	–	14.9
Parr, 2023	High-level	Medium-impact	Tennis	Ball Games	6	15	40%	–	–	–	–	–	–	–	–	–	–	–	–	14.9
Parr, 2023	High-level	Medium-impact	Lacrosse	Ball Games	4	18	22%	–	–	–	–	–	–	–	–	–	–	–	–	14.9
Parr, 2023	High-level	High-impact	Volleyball	Ball Games	3	19	16%	–	–	–	–	–	–	–	–	–	–	–	–	14.9
Parr, 2023	High-level	Medium-impact	Soccer	Ball Games	8	37	22%	–	–	–	–	–	–	–	–	–	–	–	–	14.9
Parr, 2023	High-level	Medium-impact	Softball	Ball Games	16	41	39%	–	–	–	–	–	–	–	–	–	–	–	–	14.9
Parr, 2023	High-level	Medium-impact	Cross Country	Endurance	6	9	67%	–	–	–	–	–	–	–	–	–	–	–	–	14.9
Parr, 2023	High-level	Low-impact	Swimming	Endurance	3	20	15%	–	–	–	–	–	–	–	–	–	–	–	–	14.9
Parr, 2023	High-level	Medium-impact	Track and field	Endurance	15	26	58%	–	–	–	–	–	–	–	–	–	–	–	–	14.9
Parr, 2023	High-level	Low-impact	Golf	Technical	1	6	17%	–	–	–	–	–	–	–	–	–	–	–	–	14.9
Patrizzi, 2014	Amateur	High-impact	Aerobics	Aesthetic	15	36	42%	–	–	–	–	–	–	–	–	–	15	36	–	–
Patrizzi, 2014	Amateur	Low-impact	Swimming	Endurance	9	36	25%	–	–	–	–	–	–	–	–	–	22	36	–	–
Patrizzi, 2014	Amateur	High-impact	Muscle training	Weight	22	36	61%	–	–	–	–	–	–	–	–	–	9	36	–	–
Pisani, 2022	Amateur	High-impact	CrossFit	Weight	200	616	32%	–	–	–	–	–	–	–	–	–	–	–	–	–
Poświata, 2014	Professional	Medium-impact	Athletics	Endurance	34	55	62%	23	55	42%	16	55	29%	–	–	–	–	–	–	–
Poświata, 2014	Professional	Low-impact	Cross-country skiing	Endurance	33	57	58%	28	57	49%	17	57	30%	–	–	–	–	–	–	–
Sandwith, 2021	High-level	Medium-impact	Rugby	Ball Games	51	95	54%	–	–	–	–	–	–	–	–	–	–	–	–	14.9
Sebastián Rico, 2024	Professional	Medium-impact	Soccer	Ball Games	82	235	35%	61	235	26%	23	235	10%	11	235	5%	–	–	–	–
Tarczewska, 2024	Amateur	High-impact	Volleyball	Ball Games	5	10	50%	–	–	–	–	–	–	–	–	–	–	–	–	–
Tarczewska, 2024	High-level	High-impact	Basketball	Ball Games	6	11	55%	–	–	–	–	–	–	–	–	–	–	–	–	–
Tarczewska, 2024	High-level	Medium-impact	Athletics	Endurance	6	25	24%	–	–	–	–	–	–	–	–	–	–	–	–	–
Tarczewska, 2024	Amateur	High-impact	Strength sports	Weight	10	15	67%	–	–	–	–	–	–	–	–	–	–	–	–	–
Valastro, 2025	NR	Medium-impact	Rugby	Ball Games	10	37	27%	–	–	–	–	–	–	–	–	–	10	37	–	–
Winder, 2023	Professional	High-impact	Dance	Aesthetic	60	208	29%	110	208	53%	66	208	32%	–	–	–	113	208	–	25

NR: Not reported; NC: Not classified; UI: Urinary incontinence; SUI: Stress UI; UUI: Urgency UI; MUI: Mixed UI; EXUI; Exercise-related/exertional UI.
